# Gemcitabine and Arabinosylcytosin Pharmacogenomics: Genome-Wide Association and Drug Response Biomarkers

**DOI:** 10.1371/journal.pone.0007765

**Published:** 2009-11-09

**Authors:** Liang Li, Brooke L. Fridley, Krishna Kalari, Gregory Jenkins, Anthony Batzler, Richard M. Weinshilboum, Liewei Wang

**Affiliations:** 1 Molecular Pharmacology and Experimental Therapeutics, Mayo Clinic, Rochester, Minnesota, United States of America; 2 Health Sciences Research, Mayo Clinic, Rochester, Minnesota, United States of America; National Cancer Institute, United States of America

## Abstract

Cancer patients show large individual variation in their response to chemotherapeutic agents. Gemcitabine (dFdC) and AraC, two cytidine analogues, have shown significant activity against a variety of tumors. We previously used expression data from a lymphoblastoid cell line-based model system to identify genes that might be important for the two drug cytotoxicity. In the present study, we used that same model system to perform a genome-wide association (GWA) study to test the hypothesis that common genetic variation might influence both gene expression and response to the two drugs. Specifically, genome-wide single nucleotide polymorphisms (SNPs) and mRNA expression data were obtained using the Illumina 550K® HumanHap550 SNP Chip and Affymetrix U133 Plus 2.0 GeneChip, respectively, for 174 ethnically-defined “Human Variation Panel” lymphoblastoid cell lines. Gemcitabine and AraC cytotoxicity assays were performed to obtain IC_50_ values for the cell lines. We then performed GWA studies with SNPs, gene expression and IC_50_ of these two drugs. This approach identified SNPs that were associated with gemcitabine or AraC IC_50_ values and with the expression regulation for 29 genes or 30 genes, respectively. One SNP in *IQGAP2* (rs3797418) was significantly associated with variation in both the expression of multiple genes and gemcitabine and AraC IC_50_. A second SNP in *TGM3* (rs6082527) was also significantly associated with multiple gene expression and gemcitabine IC50. To confirm the association results, we performed siRNA knock down of selected genes with expression that was associated with rs3797418 and rs6082527 in tumor cell and the knock down altered gemcitabine or AraC sensitivity, confirming our association study results. These results suggest that the application of GWA approaches using cell-based model systems, when combined with complementary functional validation, can provide insights into mechanisms responsible for variation in cytidine analogue response.

## Introduction

The application of high-throughput genomic techniques makes it possible to identify variation across the genome that may help to predict clinical response to drug treatment [1]–[5]. Wide variations in response to many antineoplastic drugs exist in the clinic. Therefore, it is crucial to identify biomarkers that will help make it possible to maximizing efficacy while minimize drug-related toxicity. One possible explanation for variation in response to chemotherapy is genetic variation that influences gene expression and, ultimately, impacts variation in drug response phenotypes [6]–[8]. Most previous studies have focused on biomarkers in tumor tissue and their relationship with therapeutic response [9]–[15]. While identifying genetic biomarkers in the tumor is important for understanding tumorigenesis and for predicting prognosis and treatment response, individual variation in germline DNA can also play an important role in drug response. Gemcitabine and AraC are two cytidine analogues that are widely used to treat a variety of cancers [16]–[19]. Only 20% of the pancreatic cancer patients treated with gemcitabine have life expectancy over 1 year. Many side effects such as GI and hematological side effects are associated with the use of these drugs. Therefore, it would be important to identify biomarkers that might help to select responsive patients and avoid side effects during treatment with these drugs.

In a present study, we took advantage of ethnically diverse Coriell “Human Variation Panel” lymphoblastoid cell lines to test the hypothesis that genetic variation across the genome in germline DNA might impact gemcitabine and AraC cytotoxicity. These cell lines were obtained from three different ethnic groups: Caucasian-Americans, African-Americans and Han Chinese-Americans. Although they are Epstein-Barr virus (EBV) transformed cell lines with expression profiles distinctive from those in tumor cells, these cell lines provide an opportunity to query the impact of common genomic variation on drug response [5], [20]–[25]. Specifically, we have now used these cell lines to perform GWA experiments designed to identify genetic variation that might be associated with variation in gene expression and then, subsequently, with variation in gemcitabine and AraC IC_50_ values. We identified 19 and 30 SNPs associated with gemcitabine and AraC IC_50_ that were also associated with the expression of 29 and 30 genes, respectively. At the same time, this variation in gene expression was associated with IC_50_ values for the two drug. We then focused on 2 SNPs with unknown function in the intron and 5′-flank region (FR) of a gene encoding the IQ motif containing GTPase activating protein 2 (*IQGAP2*),(rs3797418) and a gene encoding transglutaminase 3 (*TGM3*), (rs6082527), respectively. *IQGAP2* (rs3797418) was significantly associated with IC_50_ for both drugs and *TGM3* (rs6082527) was significantly associated with the expression of multiple genes and with gemcitabine IC_50_ values. These two SNPs are not in linkage disequilibrium (LD) with each other. Because gemcitabine is used to treat a variety of solid tumors including pancreatic cancer, in order to validate this association obtained using lymphoblastoid cell lines, we selected two pancreatic tumor cell lines to perform siRNA knock down of three selected genes (*MGMT*, *VAV3* and *GPM6A*) with expression levels that were associated with either or both of the rs3797418 and rs6082527 SNPs. Those results indicated that down-regulation of VAV3 and GPM6A altered gemcitabine or AraC response, as predicted by the association studies. In addition, we also took the advantage of the fact that we had information with regard to SNPs for all of the genes involved in the cytidine analogue activation and deactivation pathway, and performed SNP expression as well as drug cytotoxicity analyses with those genes. These results suggest that a genome-wide approach can help to identify genetic variation that might be important for variation in drug response through the trans-regulation of the expression of multiple genes.

## Results

### Genome-Wide SNP vs. Gemcitabine IC_50_ Association Study

**Figure 1:**
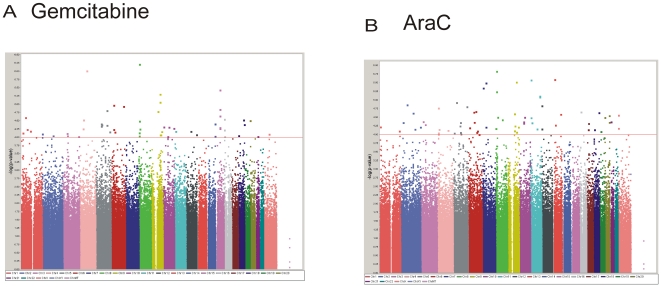
Genome-wide SNP association with drug IC_50_. (A). Genome-wide SNP-gemcitabine IC_50_ association data. (B). Genome-wide SNP-AraC IC_50_ association data. Genome-wide SNPs were obtained for all 171 cell lines using Illumina 550K Beadchips. Genome-wide SNP association analysis was then performed with gemcitabine IC_50_ as the drug-response phenotype (n = 171). The y-axis is the −log10 (p-value) for the SNP. SNPs are plotted on the x-axis on the basis of their chromosomal locations. The “cut-off” p-value of 0.001 is highlighted with a red line.

**Figure 2:**
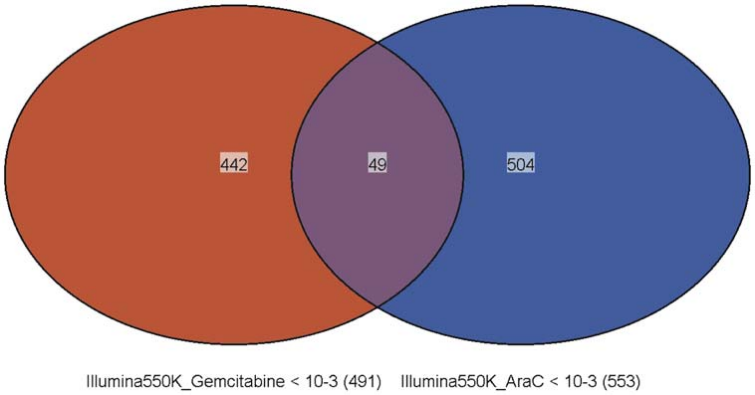
Top candidate SNPs that overlapped between gemcitabine and AraC. 49 SNPs were common (p<0.001) among 442 unique SNPs associated with gemcitabine and 504 associated with AraC.

A total of 561,464 SNPs were genotyped on the Illumina 550K SNP array and were subjected to quality control prior to performing the association study. As a result, we first removed the 8,222 SNPs that had call rate <95% as well as the 33,564 SNPs with Minor Allele Frequencies (MAFs) <5%. Finally, we removed 4,639 SNPs that deviated from Hardy-Weinberg Equilibrium (HWE) using a stringent threshold of p<0.001. Therefore, a total of 515,039 SNPs were used in a genome-wide SNP analyses for 171 cell lines. **Figure 1** depicts graphically and **Table S1 and S2** list the 492 SNPs associated with gemcitabine IC_50_ and the 553 SNPs associated with AraC IC_50_ with p-values <10^−3^. 58 and 68 SNPs among these 492 and 553 SNPs had p-values <10^−4^, respectively. These 58 and 68 SNPs were in or near 47 or 59 unique genes on the basis of the annotation provided by Illumina, genome build 36.1. 14 of those 58 SNPs were in 3′-flanking regions, 19 in 5′-flanking regions, 23 in intragenic regions of the closest gene, while only two were in the coding regions for the *PIGB* and *HLA-DRA* genes. The top three SNPs from the GWAS, rs4272382 (p = 6.14×10^−7^), rs3775182 (9.67×10^−7^) and rs2290344 (3.65×10^−6^), were in genes encoding claudin 23, MAPK10 and PIGB. For the 68 top SNPs associated with AraC IC_50_ in 59 unique genes, 28 SNPs were in 3′-flanking regions, 17 in 5′-flanking regions and 23 in introns. None were in coding regions. The top three SNPs for AraC were rs2604376 (p = 1.84×10^−6^), rs9512755 (p = 2.54×10^−6^) and rs2595500 (p = 2.63×10^−6^) in the *TUSC3*, *LNX2* and *ZNF215* genes. When we compared gemcitabine and AraC, among the top SNPs that were associated with drug IC_50_ values (p<0.001), only 49 were common to both drugs (**Table S3 and** **Figure 2**).

### SNP and Gene Expression Association

Among the above associated 492 associated with gemcitabine IC_50_, we observed that 100 unique SNPs, located within or near 78 genes on the basis of the Illumina annotation were associated with variation in the expression of 296 probesets (220 unique genes) with a cutoff of p-values <10^−6^. Among the 553 SNPs associated with AraC IC_50_, 140 unique SNPs within or near 123 genes were associated with variation in expression of 843 (563 unique genes) expression probesets (**Table S4 and S5**). Only 12 out of the 100 SNPs or 32 out of the 140 SNPs were in cis-regulatory regions for gemcitabine and AraC, respectively, defined as 5 Mb on either side of the gene, while the rest of the SNPs were localized in trans-regulatory regions, consistent with previous findings [5], [26]–[28].

### Gene Expression and IC_50_ Association

**Figure 3:**
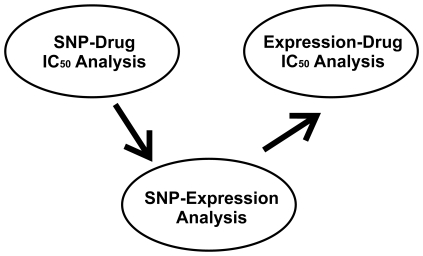
Strategy applied in these studies. A genome-wide SNP association study was performed with a drug-related phenotype, gemcitabine or AraC cytotoxicity (IC_50_). SNPs associated with cytotoxicity (p<10^−3^) were used to perform an association study with 54,000 expression probesets to identify SNPs that were associated with both gene expression (p<10^−6^) and with gemcitabine or AraC IC_50_. Finally, an association study was performed with gene expression and gemcitabine or AraC IC_50_ to identify SNPs that might be associated with drug IC_50_ values through an influence on gene expression.

Among the 296 expression probesets associated with the 100 unique SNPs for gemcitabine, we determined that 35 expression probesets were also associated with gemcitabine IC_50_ values (p<0.001). These 35 probesets represented 29 genes. By performing the same analysis for AraC, we identified 60 expression probesets that represented 54 genes which were associated with AraC IC_50_ values (p<0.001). **Figure 3** showed our integrated analysis strategy, a strategy that included SNP, gemcitabine and gene expression data. This analysis resulted in the identification of 19 SNPs close to or within 16 genes that were associated with gemcitabine IC_50_ values through an influence on the expression of 29 genes represented by 35 expression probesets (**Table 1**). The same type of analysis resulted in the identification of 30 SNPs close to or within 30 genes that were associated with AraC IC_50_ values through an influence on the expression of 54 genes represented by 60 expression probesets (**Table 2**). Among the top candidate SNPs, rs3797418 (A/C), a SNP located in an intron of *IQGAP2*, was significantly associated with the expression of multiple genes and IC_50_ values for both drug. Therefore, this SNP and gene were selected for further functional verification. In addition, a second SNP, rs6082527 (A/G), located in the 5′-FR of *TGM3*, which was only associated with gemcitabine IC_50_ values, was also selected for further functional study because of its significant association with the expression of multiple genes, and these genes were also significantly associated with gemcitabine cytotoxicity, with p values ranging from 10^−4^ to 10^−8^ (**Tables 1** **and** **2**). Although the SNP in *TGM3* was not significantly associated with AraC IC_50_ values, given the similarity between these two drugs, we also performed functional studies for *TGM3* and its downstream gene *GPM6A* with AraC. Specifically, rs3797418 was associated with the expression of 10 individual genes for gemcitabine and 7 for AraC. rs6082527 was associated with 6 probesets that targeted 5 individual genes.

**Table 1:** Top candidate SNPs and probe sets that were associated with gemcitabine IC_50_.

SNPs					Probe sets			SNP vs Expression		Expression vs IC50		SNP vs IC50	
rsID	Chr	Closest gene	SNP Location	MAF	Probeset	Chr	Gene Symbol	r-value	p-value	r-value	p-value	r-value	p-value
rs1423300	5	ADAMTS12	intron	0.526	209303_at	5	NDUFS4	−0.40	2.00E-07	0.32	2.27E-05	−0.28	3.57E-04
rs2118524	12	BTG1	flanking_5UTR	0.088	219104_at	11	RNF141	0.39	5.74E-07	−0.33	1.07E-05	−0.26	9.95E-04
rs2118524	12	BTG1	flanking_5UTR	0.088	231186_at	14	FLJ43390///LOC100128793	0.39	5.95E-07	−0.29	1.09E-04	−0.26	9.95E-04
rs1809180	15	CHSY1	flanking_3UTR	0.173	201462_at	7	SCRN1	−0.41	8.28E-08	0.34	6.47E-06	−0.27	9.12E-04
rs453169	16	FLJ32252	flanking_3UTR	0.056	231851_at	1	RAVER2	0.45	3.23E-09	−0.30	6.63E-05	−0.27	7.79E-04
rs11215406	11	IGSF4	intron	0.14	1556095_at	15	UNC13C	0.39	3.22E-07	−0.32	1.57E-05	−0.27	6.70E-04
rs11215406	11	IGSF4	intron	0.14	1569969_a_at	15	UNC13C	0.38	9.91E-07	−0.30	5.46E-05	−0.27	6.70E-04
**rs3797418**	**5**	**IQGAP2**	**intron**	**0.102**	**203726_s_at**	**18**	**LAMA3**	**0.41**	**9.07E-08**	**−0.44**	**1.23E-09**	**−0.27**	**8.11E-04**
**rs3797418**	**5**	**IQGAP2**	**intron**	**0.102**	**204105_s_at**	**7**	**NRCAM**	**0.43**	**1.65E-08**	**−0.28**	**1.75E-04**	**−0.27**	**8.11E-04**
**rs3797418**	**5**	**IQGAP2**	**intron**	**0.102**	**204880_at**	**10**	**MGMT**	**−0.41**	**1.22E-07**	**0.40**	**5.49E-08**	**−0.27**	**8.11E-04**
**rs3797418**	**5**	**IQGAP2**	**intron**	**0.102**	**205925_s_at**	**1**	**RAB3B**	**0.39**	**3.94E-07**	**−0.29**	**1.35E-04**	**−0.27**	**8.11E-04**
**rs3797418**	**5**	**IQGAP2**	**intron**	**0.102**	**205931_s_at**	**7**	**CREB5**	**0.38**	**9.37E-07**	**−0.26**	**5.71E-04**	**−0.27**	**8.11E-04**
**rs3797418**	**5**	**IQGAP2**	**intron**	**0.102**	**210058_at**	**6**	**MAPK13**	**0.39**	**3.20E-07**	**−0.27**	**3.09E-04**	**−0.27**	**8.11E-04**
**rs3797418**	**5**	**IQGAP2**	**intron**	**0.102**	**210059_s_at**	**6**	**MAPK13**	**0.46**	**1.29E-09**	**−0.31**	**4.70E-05**	**−0.27**	**8.11E-04**
**rs3797418**	**5**	**IQGAP2**	**intron**	**0.102**	**213056_at**	**3**	**FRMD4B**	**0.39**	**5.54E-07**	**−0.30**	**6.37E-05**	**−0.27**	**8.11E-04**
**rs3797418**	**5**	**IQGAP2**	**intron**	**0.102**	**216959_x_at**	**7**	**NRCAM**	**0.43**	**1.54E-08**	**−0.29**	**9.36E-05**	**−0.27**	**8.11E-04**
**rs3797418**	**5**	**IQGAP2**	**intron**	**0.102**	**218651_s_at**	**15**	**LARP6**	**0.40**	**2.39E-07**	**−0.31**	**3.71E-05**	**−0.27**	**8.11E-04**
**rs3797418**	**5**	**IQGAP2**	**intron**	**0.102**	**218806_s_at**	**1**	**VAV3**	**0.41**	**8.14E-08**	**−0.37**	**5.79E-07**	**−0.27**	**8.11E-04**
**rs3797418**	**5**	**IQGAP2**	**intron**	**0.102**	**218807_at**	**1**	**VAV3**	**0.49**	**9.16E-11**	**−0.39**	**1.49E-07**	**−0.27**	**8.11E-04**
**rs3797418**	**5**	**IQGAP2**	**intron**	**0.102**	**227123_at**	**1**	**RAB3B**	**0.47**	**4.56E-10**	**−0.26**	**4.85E-04**	**−0.27**	**8.11E-04**
**rs3797418**	**5**	**IQGAP2**	**intron**	**0.102**	**232280_at**	**14**	**SLC25A29**	**0.39**	**3.24E-07**	**−0.35**	**2.99E-06**	**−0.27**	**8.11E-04**
rs2290344	15	PIGB	coding	0.254	242760_x_at	15	PIGB	−0.41	1.43E-07	−0.29	8.98E-05	0.36	3.65E-06
rs8024695	15	PIGB	intron	0.287	242760_x_at	15	PIGB	−0.38	7.05E-07	−0.29	8.98E-05	0.34	1.43E-05
rs12188464	5	PIK3R1	flanking_3UTR	0.459	201334_s_at	11	ARHGEF12	0.38	9.03E-07	−0.34	5.35E-06	−0.32	4.57E-05
rs7713001	5	PIK3R1	flanking_3UTR	0.459	201334_s_at	11	ARHGEF12	0.38	9.03E-07	−0.34	5.35E-06	−0.32	4.57E-05
rs6625401	23	PJA1	flanking_3UTR	0.351	214908_s_at	7	TRRAP	0.39	4.31E-07	−0.27	3.28E-04	−0.31	8.03E-05
rs2217881	1	PLD5	intron	0.08	231186_at	14	FLJ43390///LOC100128793	0.44	1.46E-08	−0.29	1.09E-04	−0.29	3.35E-04
rs7313937	12	PTPRR	intron	0.088	211572_s_at	20	SLC23A2	0.41	8.23E-08	−0.33	7.50E-06	−0.27	7.17E-04
rs4896870	6	RAB32	flanking_5UTR	0.117	223194_s_at	6	SLC22A23	0.45	2.92E-09	−0.29	1.24E-04	−0.27	6.66E-04
rs4896870	6	RAB32	flanking_5UTR	0.117	242629_at	1	---	0.40	2.83E-07	−0.28	2.21E-04	−0.27	6.66E-04
rs4896870	6	RAB32	flanking_5UTR	0.117	47560_at	19	LPHN1	0.38	9.73E-07	−0.28	1.73E-04	−0.27	6.66E-04
rs907609	11	SYT8	coding	0.082	205925_s_at	1	RAB3B	0.38	6.49E-07	−0.29	1.35E-04	−0.28	4.12E-04
**rs6082527**	**20**	**TGM3**	**flanking_5UTR**	**0.058**	**204880_at**	**10**	**MGMT**	**−0.41**	**8.06E-08**	**0.40**	**5.49E-08**	**−0.28**	**4.86E-04**
**rs6082527**	**20**	**TGM3**	**flanking_5UTR**	**0.058**	**209469_at**	**4**	**GPM6A**	**0.42**	**4.67E-08**	**−0.35**	**2.08E-06**	**−0.28**	**4.86E-04**
**rs6082527**	**20**	**TGM3**	**flanking_5UTR**	**0.058**	**209470_s_at**	**4**	**GPM6A**	**0.40**	**1.99E-07**	**−0.34**	**6.37E-06**	**−0.28**	**4.86E-04**
**rs6082527**	**20**	**TGM3**	**flanking_5UTR**	**0.058**	**224325_at**	**10**	**FZD8**	**0.41**	**1.01E-07**	**−0.30**	**5.69E-05**	**−0.28**	**4.86E-04**
**rs6082527**	**20**	**TGM3**	**flanking_5UTR**	**0.058**	**224499_s_at**	**12**	**AICDA**	**−0.40**	**2.07E-07**	**0.31**	**3.71E-05**	**−0.28**	**4.86E-04**
**rs6082527**	**20**	**TGM3**	**flanking_5UTR**	**0.058**	**225784_s_at**	**23**	**KIAA1166**	**−0.43**	**2.33E-08**	**0.28**	**2.41E-04**	**−0.28**	**4.86E-04**
rs1153942	1	TP53BP2	intron	0.118	218801_at	13	UGCGL2	−0.45	3.26E-09	−0.25	8.80E-04	0.27	9.38E-04
rs612578	19	ZNF626	intron	0.24	202092_s_at	16	ARL2BP	0.39	3.50E-07	−0.38	2.62E-07	−0.29	3.33E-04
rs11666947	19	ZNF626	flanking_3UTR	0.06	231186_at	14	FLJ43390///LOC100128793	0.41	1.91E-07	−0.29	1.09E-04	−0.28	6.40E-04

35 probe sets (29 unique genes) associated with 19 SNPs (16 unique SNP-nearby genes) were significantly correlated with gemcitabine IC_50_. Genes that were associated with the two SNPs in *IQGAP2* and *TGM3* are bolded. p-value for each association are indicated. R values represent the correlation coefficient for each association.

**Table 2:** Top candidate SNPs and probe sets that were associated with AraC IC_50_.

SNPs					Probesets			SNP vs Expression(54K)		Expression (54K) vs GI_50_		SNP vs GI_50_	
rsID	Chr	Closest Gene	SNP Location	MAF	Probeset	Chr	Gene Symbol	r-value	p-value	r-value	p-value	r-value	p-value
rs11215406	11	IGSF4	intron	0.139	1556095_at	15	UNC13C	0.394	3.22E-07	−0.351	7.64E-06	−0.354	1.60E-06
rs11215406	11	IGSF4	intron	0.139	1569969_a_at	15	UNC13C	0.379	9.91E-07	−0.351	7.64E-06	−0.332	7.56E-06
rs10208516	2	CTNNA2	flanking_3UTR	0.052	214018_at	12	GRIP1	0.410	9.73E-08	0.342	1.37E-05	0.270	3.23E-04
rs10208516	2	CTNNA2	flanking_3UTR	0.052	1565812_at	5	TRIM36	0.414	7.34E-08	0.342	1.37E-05	0.268	3.53E-04
rs10208516	2	CTNNA2	flanking_3UTR	0.052	205088_at	23	MAMLD1	0.409	1.02E-07	0.342	1.37E-05	0.263	4.64E-04
rs10208516	2	CTNNA2	flanking_3UTR	0.052	203443_at	11	EML3	0.422	3.57E-08	0.342	1.37E-05	0.255	6.84E-04
rs10208516	2	CTNNA2	flanking_3UTR	0.052	206696_at	23	GPR143	0.413	7.89E-08	0.342	1.37E-05	0.248	9.80E-04
rs888468	12	FGF6	flanking_3UTR	0.182	211572_s_at	20	SLC23A2	0.405	1.43E-07	−0.341	1.45E-05	−0.264	4.25E-04
rs11136070	8	DUSP4	flanking_5UTR	0.052	209756_s_at	2	MYCN	0.421	4.04E-08	0.328	3.15E-05	0.312	2.73E-05
rs11136070	8	DUSP4	flanking_5UTR	0.052	1553755_at	19	NXNL1	0.395	3.14E-07	0.328	3.15E-05	0.312	2.82E-05
rs11136070	8	DUSP4	flanking_5UTR	0.052	1554782_at	2	C2orf19	0.453	2.64E-09	0.328	3.15E-05	0.304	4.58E-05
rs11136070	8	DUSP4	flanking_5UTR	0.052	242071_x_at	10	ITGA8	0.411	8.62E-08	0.328	3.15E-05	0.276	2.22E-04
rs11136070	8	DUSP4	flanking_5UTR	0.052	213417_at	17	TBX2	0.475	3.21E-10	0.328	3.15E-05	0.274	2.51E-04
rs11136070	8	DUSP4	flanking_5UTR	0.052	240729_at	3	C3orf44	0.382	7.93E-07	0.328	3.15E-05	0.271	3.04E-04
rs11136070	8	DUSP4	flanking_5UTR	0.052	214018_at	12	GRIP1	0.490	7.06E-11	0.328	3.15E-05	0.270	3.23E-04
rs11136070	8	DUSP4	flanking_5UTR	0.052	1565812_at	5	TRIM36	0.461	1.25E-09	0.328	3.15E-05	0.268	3.53E-04
rs11136070	8	DUSP4	flanking_5UTR	0.052	1569002_x_at	8	BMP1	0.404	1.50E-07	0.328	3.15E-05	0.266	3.80E-04
rs11136070	8	DUSP4	flanking_5UTR	0.052	205947_s_at	7	VIPR2	0.446	4.84E-09	0.328	3.15E-05	0.265	4.20E-04
rs11136070	8	DUSP4	flanking_5UTR	0.052	205088_at	23	MAMLD1	0.499	2.89E-11	0.328	3.15E-05	0.263	4.64E-04
rs11136070	8	DUSP4	flanking_5UTR	0.052	235820_at	12	LOC100130219	0.430	1.89E-08	0.328	3.15E-05	0.262	4.90E-04
rs11136070	8	DUSP4	flanking_5UTR	0.052	215527_at	6	KHDRBS2	0.428	2.17E-08	0.328	3.15E-05	0.259	5.71E-04
rs11136070	8	DUSP4	flanking_5UTR	0.052	244036_at	NA	---	0.480	2.01E-10	0.328	3.15E-05	0.257	6.26E-04
rs11136070	8	DUSP4	flanking_5UTR	0.052	203443_at	11	EML3	0.438	9.32E-09	0.328	3.15E-05	0.255	6.84E-04
rs11136070	8	DUSP4	flanking_5UTR	0.052	230249_at	8	KHDRBS3	0.423	3.49E-08	0.328	3.15E-05	0.255	6.96E-04
rs11136070	8	DUSP4	flanking_5UTR	0.052	215953_at	1	DKFZP564C196	0.387	5.72E-07	0.328	3.15E-05	0.252	8.12E-04
rs11136070	8	DUSP4	flanking_5UTR	0.052	206696_at	23	GPR143	0.455	2.19E-09	0.328	3.15E-05	0.248	9.80E-04
rs970084	20	SNPH	flanking_5UTR	0.081	222895_s_at	14	BCL11B	0.416	5.93E-08	−0.327	3.21E-05	−0.271	3.03E-04
rs970084	20	SNPH	flanking_5UTR	0.081	219528_s_at	14	BCL11B	0.390	4.46E-07	−0.327	3.21E-05	−0.269	3.36E-04
rs12412561	10	PCDH15	flanking_3UTR	0.069	205924_at	1	RAB3B	0.416	6.00E-08	−0.321	4.68E-05	−0.265	4.15E-04
rs12128558	1	NPHP4	flanking_3UTR	0.055	1569419_at	NA	---	0.441	7.30E-09	0.317	5.99E-05	0.333	6.90E-06
rs12128558	1	NPHP4	flanking_3UTR	0.055	234896_at	NA	---	0.465	8.89E-10	0.317	5.99E-05	0.259	5.49E-04
rs11876487	18	GATA6	flanking_3UTR	0.069	205947_s_at	7	VIPR2	0.406	1.28E-07	0.313	7.16E-05	0.265	4.20E-04
rs11876487	18	GATA6	flanking_3UTR	0.069	215953_at	1	DKFZP564C196	0.403	1.68E-07	0.313	7.16E-05	0.252	8.12E-04
rs613359	4	LOC132321	flanking_3UTR	0.061	1554782_at	2	C2orf19	0.402	1.78E-07	0.313	7.52E-05	0.304	4.58E-05
rs613359	4	LOC132321	flanking_3UTR	0.061	206401_s_at	17	MAPT	0.387	5.62E-07	0.313	7.52E-05	0.297	6.81E-05
rs613359	4	LOC132321	flanking_3UTR	0.061	230666_at	7	tcag7.1238	0.401	1.95E-07	0.313	7.52E-05	0.288	1.19E-04
rs613359	4	LOC132321	flanking_3UTR	0.061	242071_x_at	10	ITGA8	0.385	6.44E-07	0.313	7.52E-05	0.276	2.22E-04
rs613359	4	LOC132321	flanking_3UTR	0.061	213417_at	17	TBX2	0.436	1.17E-08	0.313	7.52E-05	0.274	2.51E-04
rs613359	4	LOC132321	flanking_3UTR	0.061	214018_at	12	GRIP1	0.451	3.20E-09	0.313	7.52E-05	0.270	3.23E-04
rs613359	4	LOC132321	flanking_3UTR	0.061	1565812_at	5	TRIM36	0.421	4.11E-08	0.313	7.52E-05	0.268	3.53E-04
rs613359	4	LOC132321	flanking_3UTR	0.061	1569002_x_at	8	BMP1	0.407	1.27E-07	0.313	7.52E-05	0.266	3.80E-04
rs613359	4	LOC132321	flanking_3UTR	0.061	205947_s_at	7	VIPR2	0.461	1.20E-09	0.313	7.52E-05	0.265	4.20E-04
rs613359	4	LOC132321	flanking_3UTR	0.061	205088_at	23	MAMLD1	0.438	1.00E-08	0.313	7.52E-05	0.263	4.64E-04
rs613359	4	LOC132321	flanking_3UTR	0.061	235820_at	12	LOC100130219	0.398	2.52E-07	0.313	7.52E-05	0.262	4.90E-04
rs613359	4	LOC132321	flanking_3UTR	0.061	215527_at	6	KHDRBS2	0.403	1.62E-07	0.313	7.52E-05	0.259	5.71E-04
rs613359	4	LOC132321	flanking_3UTR	0.061	215953_at	1	DKFZP564C196	0.426	2.72E-08	0.313	7.52E-05	0.252	8.12E-04
rs613359	4	LOC132321	flanking_3UTR	0.061	224168_at	NA	TXNDC2	0.383	7.59E-07	0.313	7.52E-05	0.251	8.19E-04
rs613359	4	LOC132321	flanking_3UTR	0.061	206696_at	23	GPR143	0.379	9.67E-07	0.313	7.52E-05	0.248	9.80E-04
rs10780183	9	EHMT1	flanking_5UTR	0.127	225532_at	18	CABLES1	−0.386	5.90E-07	−0.302	1.36E-04	0.271	2.92E-04
rs7612441	3	LRIG1	flanking_5UTR	0.145	236565_s_at	15	LARP6	0.445	5.28E-09	−0.298	1.69E-04	−0.274	2.49E-04
**rs3797418**	**5**	**IQGAP2**	**intron**	**0.101**	**204880_at**	**10**	**MGMT**	**−0.407**	**1.22E-07**	**−0.296**	**1.85E-04**	**0.332**	**7.44E-06**
**rs3797418**	**5**	**IQGAP2**	**intron**	**0.101**	**204105_s_at**	**7**	**NRCAM**	**0.432**	**1.65E-08**	**−0.296**	**1.85E-04**	**−0.297**	**6.80E-05**
**rs3797418**	**5**	**IQGAP2**	**intron**	**0.101**	**218651_s_at**	**15**	**LARP6**	**0.398**	**2.39E-07**	**−0.296**	**1.85E-04**	**−0.289**	**1.11E-04**
**rs3797418**	**5**	**IQGAP2**	**intron**	**0.101**	**210058_at**	**6**	**MAPK13**	**0.394**	**3.20E-07**	**−0.296**	**1.85E-04**	**−0.284**	**1.48E-04**
**rs3797418**	**5**	**IQGAP2**	**intron**	**0.101**	**218807_at**	**1**	**VAV3**	**0.488**	**9.16E-11**	**−0.296**	**1.85E-04**	**−0.279**	**1.95E-04**
**rs3797418**	**5**	**IQGAP2**	**intron**	**0.101**	**203726_s_at**	**18**	**LAMA3**	**0.411**	**9.07E-08**	**−0.296**	**1.85E-04**	**−0.277**	**2.15E-04**
**rs3797418**	**5**	**IQGAP2**	**intron**	**0.101**	**216959_x_at**	**7**	**NRCAM**	**0.433**	**1.54E-08**	**−0.296**	**1.85E-04**	**−0.274**	**2.52E-04**
**rs3797418**	**5**	**IQGAP2**	**intron**	**0.101**	**210059_s_at**	**6**	**MAPK13**	**0.461**	**1.29E-09**	**−0.296**	**1.85E-04**	**−0.260**	**5.40E-04**
**rs3797418**	**5**	**IQGAP2**	**intron**	**0.101**	**213056_at**	**3**	**FRMD4B**	**0.387**	**5.54E-07**	**−0.296**	**1.85E-04**	**−0.259**	**5.45E-04**
**rs3797418**	**5**	**IQGAP2**	**intron**	**0.101**	**218806_s_at**	**1**	**VAV3**	**0.412**	**8.14E-08**	**−0.296**	**1.85E-04**	**−0.248**	**9.46E-04**
rs17637119	3	CADPS	intron	0.069	204880_at	10	MGMT	−0.382	8.06E-07	−0.296	1.88E-04	0.332	7.44E-06
rs17637119	3	CADPS	intron	0.069	204866_at	23	PHF16	0.392	3.94E-07	−0.296	1.88E-04	−0.311	3.00E-05
rs17637119	3	CADPS	intron	0.069	204105_s_at	7	NRCAM	0.421	4.15E-08	−0.296	1.88E-04	−0.297	6.80E-05
rs17637119	3	CADPS	intron	0.069	218807_at	1	VAV3	0.451	3.04E-09	−0.296	1.88E-04	−0.279	1.95E-04
rs17637119	3	CADPS	intron	0.069	203726_s_at	18	LAMA3	0.404	1.54E-07	−0.296	1.88E-04	−0.277	2.15E-04
rs17637119	3	CADPS	intron	0.069	216959_x_at	7	NRCAM	0.416	5.89E-08	−0.296	1.88E-04	−0.274	2.52E-04
rs17637119	3	CADPS	intron	0.069	222548_s_at	2	MAP4K4	0.382	7.91E-07	−0.296	1.88E-04	−0.273	2.63E-04
rs17637119	3	CADPS	intron	0.069	218181_s_at	2	MAP4K4	0.381	8.73E-07	−0.296	1.88E-04	−0.271	2.94E-04
rs17637119	3	CADPS	intron	0.069	203922_s_at	23	CYBB	−0.393	3.45E-07	−0.296	1.88E-04	0.265	4.12E-04
rs17637119	3	CADPS	intron	0.069	210059_s_at	6	MAPK13	0.382	7.87E-07	−0.296	1.88E-04	−0.260	5.40E-04
rs17637119	3	CADPS	intron	0.069	213056_at	3	FRMD4B	0.379	9.66E-07	−0.296	1.88E-04	−0.259	5.45E-04
rs17637119	3	CADPS	intron	0.069	239202_at	NA	---	0.414	7.17E-08	−0.296	1.88E-04	−0.250	8.71E-04
rs17637119	3	CADPS	intron	0.069	218806_s_at	1	VAV3	0.431	1.81E-08	−0.296	1.88E-04	−0.248	9.46E-04
rs11796743	23	GSPT2	flanking_5UTR	0.061	1553755_at	19	NXNL1	0.420	4.75E-08	0.288	2.90E-04	0.312	2.82E-05
rs1408077	1	CR1	intron	0.195	1567320_at	NA	LOC57802	0.403	1.76E-07	0.283	3.85E-04	0.249	9.14E-04
rs5962901	23	MID2	intron	0.067	226529_at	7	TMEM106B	−0.380	9.95E-07	0.280	4.38E-04	−0.276	2.23E-04
rs5962901	23	MID2	intron	0.067	213417_at	17	TBX2	0.408	1.26E-07	0.280	4.38E-04	0.274	2.51E-04
rs5962901	23	MID2	intron	0.067	214018_at	12	GRIP1	0.403	1.78E-07	0.280	4.38E-04	0.270	3.23E-04
rs5962901	23	MID2	intron	0.067	1565812_at	5	TRIM36	0.413	8.17E-08	0.280	4.38E-04	0.268	3.53E-04
rs5962901	23	MID2	intron	0.067	205088_at	23	MAMLD1	0.389	5.18E-07	0.280	4.38E-04	0.263	4.64E-04
rs5962901	23	MID2	intron	0.067	215527_at	6	KHDRBS2	0.385	6.82E-07	0.280	4.38E-04	0.259	5.71E-04
rs5962901	23	MID2	intron	0.067	206696_at	23	GPR143	0.432	1.73E-08	0.280	4.38E-04	0.248	9.80E-04
rs17183491	15	WDR72	flanking_5UTR	0.263	227067_x_at	1	NOTCH2NL	−0.382	9.55E-07	0.280	4.59E-04	−0.256	6.48E-04
rs6491526	13	CLYBL	intron	0.075	214018_at	12	GRIP1	0.398	2.46E-07	0.277	4.84E-04	0.270	3.23E-04
rs6491526	13	CLYBL	intron	0.075	1569002_x_at	8	BMP1	0.382	7.76E-07	0.277	4.84E-04	0.266	3.80E-04
rs6491526	13	CLYBL	intron	0.075	210961_s_at	20	ADRA1D	0.380	9.07E-07	0.277	4.84E-04	0.254	7.25E-04
rs1669748	5	ADAMTS16	flanking_5UTR	0.462	239202_at	NA	---	0.394	3.21E-07	−0.277	4.93E-04	−0.250	8.71E-04
rs11706227	3	WNT5A	flanking_3UTR	0.061	1560397_s_at	NA	KLHL6	−0.384	6.71E-07	0.274	5.61E-04	−0.286	1.33E-04
rs9833533	3	FHIT	intron	0.058	1554782_at	2	C2orf19	0.488	9.19E-11	0.272	6.23E-04	0.304	4.58E-05
rs9833533	3	FHIT	intron	0.058	1570330_at	NA	---	0.386	5.97E-07	0.272	6.23E-04	0.284	1.42E-04
rs9833533	3	FHIT	intron	0.058	214018_at	12	GRIP1	0.424	3.25E-08	0.272	6.23E-04	0.270	3.23E-04
rs9833533	3	FHIT	intron	0.058	1565812_at	5	TRIM36	0.423	3.44E-08	0.272	6.23E-04	0.268	3.53E-04
rs9833533	3	FHIT	intron	0.058	205947_s_at	7	VIPR2	0.384	6.71E-07	0.272	6.23E-04	0.265	4.20E-04
rs9833533	3	FHIT	intron	0.058	205088_at	23	MAMLD1	0.379	9.83E-07	0.272	6.23E-04	0.263	4.64E-04
rs9833533	3	FHIT	intron	0.058	235820_at	12	LOC100130219	0.446	4.75E-09	0.272	6.23E-04	0.262	4.90E-04
rs9833533	3	FHIT	intron	0.058	215527_at	6	KHDRBS2	0.463	9.78E-10	0.272	6.23E-04	0.259	5.71E-04
rs9833533	3	FHIT	intron	0.058	203443_at	11	EML3	0.401	1.89E-07	0.272	6.23E-04	0.255	6.84E-04
rs9833533	3	FHIT	intron	0.058	230249_at	8	KHDRBS3	0.381	8.25E-07	0.272	6.23E-04	0.255	6.96E-04
rs9833533	3	FHIT	intron	0.058	215953_at	1	DKFZP564C196	0.401	1.99E-07	0.272	6.23E-04	0.252	8.12E-04
rs9833533	3	FHIT	intron	0.058	206696_at	23	GPR143	0.446	4.87E-09	0.272	6.23E-04	0.248	9.80E-04
rs13197839	6	EGFL11	flanking_3UTR	0.119	200972_at	15	TSPAN3	−0.382	8.52E-07	0.269	7.47E-04	−0.266	3.94E-04
rs5984367	23	TGIF2LX	flanking_5UTR	0.098	207278_s_at	19	CD209	0.424	3.09E-08	0.268	7.48E-04	0.289	1.10E-04
rs11770570	7	LHFPL3	intron	0.055	209756_s_at	2	MYCN	0.441	7.77E-09	0.267	7.95E-04	0.312	2.73E-05
rs11770570	7	LHFPL3	intron	0.055	230666_at	7	tcag7.1238	0.400	2.12E-07	0.267	7.95E-04	0.288	1.19E-04
rs11770570	7	LHFPL3	intron	0.055	214454_at	5	ADAMTS2	0.395	3.13E-07	0.267	7.95E-04	0.274	2.55E-04
rs11770570	7	LHFPL3	intron	0.055	214018_at	12	GRIP1	0.426	2.70E-08	0.267	7.95E-04	0.270	3.23E-04
rs11770570	7	LHFPL3	intron	0.055	1565812_at	5	TRIM36	0.456	1.98E-09	0.267	7.95E-04	0.268	3.53E-04
rs11770570	7	LHFPL3	intron	0.055	205088_at	23	MAMLD1	0.446	4.60E-09	0.267	7.95E-04	0.263	4.64E-04
rs11770570	7	LHFPL3	intron	0.055	235820_at	12	LOC100130219	0.442	6.92E-09	0.267	7.95E-04	0.262	4.90E-04
rs11770570	7	LHFPL3	intron	0.055	203443_at	11	EML3	0.424	3.05E-08	0.267	7.95E-04	0.255	6.84E-04
rs11770570	7	LHFPL3	intron	0.055	244522_at	11	SYVN1	0.407	1.19E-07	0.267	7.95E-04	0.248	9.64E-04
rs11770570	7	LHFPL3	intron	0.055	206696_at	23	GPR143	0.422	3.65E-08	0.267	7.95E-04	0.248	9.80E-04
rs11063679	12	NTF3	flanking_5UTR	0.075	205088_at	23	MAMLD1	0.461	1.19E-09	0.265	8.56E-04	0.263	4.64E-04
rs5768857	22	CELSR1	intron	0.127	209310_s_at	11	CASP4	0.381	8.40E-07	0.265	8.57E-04	0.267	3.65E-04
rs4730550	7	IFRD1	intron	0.064	218229_s_at	1	POGK	−0.401	2.18E-07	0.266	8.73E-04	−0.256	6.58E-04
rs8180101	3	GBE1	flanking_5UTR	0.081	205982_x_at	8	SFTPC	0.393	3.64E-07	0.264	8.91E-04	0.248	9.83E-04
rs772492	4	LOC285513	flanking_5UTR	0.173	213056_at	3	FRMD4B	0.379	9.81E-07	−0.264	8.98E-04	−0.259	5.45E-04
rs549467	4	RAP1GDS1	flanking_5UTR	0.069	216030_s_at	20	SEMG2	0.436	1.15E-08	0.263	9.43E-04	0.332	7.74E-06

60 probe sets (54 unique genes) associated with 30 SNPs (30 unique SNP-nearby genes) were significantly correlated with AraC IC50. Genes that were associated with SNPs in IQGAP2 are bolded for AraC, too. p-value for each association are indicated. R values represent the correlation coefficient for each association.

### Cytidine Analogue Pathway Gene Analysis

Gemcitabine and AraC share the same metabolism pathway that activates these drugs in cells. There is a total of 19 genes within this pathway (**Table S6**). Many previous pharmacogenetic studies have been focused on genetic variation in genes within this pathway, and there is evidence showing that variation in the expression of genes within this pathway can influence response to cytidine analogues [7], [29], [30]. Therefore, we took the advantage of the genome-wide SNP data and selected all SNPs within pathway genes that were present on the Illumina 550K chip to perform an analysis among SNP, expression and gemcitabine and AraC IC_50_ values for these pathway genes. A total of 749 SNPs for cytidine analogue pathway genes were present on the Illumina 550K chip. Association of gemcitabine IC_50_ with SNPs in pathway genes identified 38 SNPs within or close to 8 genes with p-values <0.05, while 23 SNPs close to 9 genes were significantly associated with AraC IC_50_ values with p-values <0.05 (**Tables 3** and **4**). To determine the association of SNPs with cis-regulation of pathway gene expression, we associated all of these SNPs with expression for all genes within the pathway. That analysis identified 24 cis-acting SNPs that regulate the expression of pathway genes with p-values <0.0001 (**Table 5**). The top three SNPs (p = 7.54×10^−33^, 1.54×10^−32^ and 2.69×10^−10^, respectively) were all mapped to the *NT5C3L1* gene, a nucleotidase family member (**Table 5**).

**Table 3:** Top 38 significant SNPs within or close to 8 pathway genes associated with gemcitabine IC_50_ values (p<0.05).

SNP	r-value	p-value	q-value	N	MAF	Chr	Position	Genome Build	Gene Symbol	Gene	Location	Location Relative to Gene
rs2037067	0.269	0.001	0.82119	170	0.471	4	1.84E+08	36.1	DCTD	NM_001012732.1	flanking_3UTR	−237660
rs3775289	0.227	0.005	0.82119	170	0.203	4	72081538	36.1	DCK	NM_000788.1	Intron	−1110
rs4308342	0.227	0.005	0.82119	170	0.203	4	72103069	36.1	DCK	NM_000788.1	Intron	−3879
rs956727	−0.218	0.007	0.82119	171	0.117	9	86036753	36.1	SLC28A3	NM_022127.1	flanking_3UTR	−46159
rs515583	0.211	0.009	0.82119	171	0.19	6	85933010	36.1	NT5E	NM_002526.1	flanking_5UTR	−283518
rs13144500	−0.205	0.011	0.82119	171	0.123	4	1.84E+08	36.1	DCTD	NM_001012732.1	flanking_3UTR	−335310
rs12500335	−0.202	0.013	0.82119	170	0.091	4	1.84E+08	36.1	DCTD	NM_001012732.1	flanking_3UTR	−103463
rs3966882	0.202	0.013	0.82119	168	0.318	6	85938190	36.1	NT5E	NM_002526.1	flanking_5UTR	−278338
rs10006225	0.200	0.013	0.82119	171	0.064	4	1.84E+08	36.1	DCTD	NM_001012732.1	flanking_3UTR	−102248
rs1932660	0.200	0.013	0.82119	171	0.234	9	86017079	36.1	SLC28A3	NM_022127.1	flanking_3UTR	−65833
rs4877822	0.200	0.013	0.82119	171	0.234	9	86021002	36.1	SLC28A3	NM_022127.1	flanking_3UTR	−61910
rs7874528	−0.198	0.014	0.82119	171	0.17	9	86020319	36.1	SLC28A3	NM_022127.1	flanking_3UTR	−62593
rs9472236	0.196	0.015	0.82119	171	0.225	6	44314728	36.1	SLC29A1	NM_004955.1	flanking_3UTR	−4872
rs1570933	0.196	0.015	0.82119	171	0.292	6	85937335	36.1	NT5E	NM_002526.1	flanking_5UTR	−279193
rs17087049	−0.194	0.017	0.82119	171	0.208	9	86104192	36.1	SLC28A3	NM_022127.1	Intron	−124
rs10780659	−0.193	0.017	0.82119	171	0.146	9	86071310	36.1	SLC28A3	NM_022127.1	flanking_3UTR	−11602
rs7173860	0.192	0.017	0.82119	171	0.465	15	83230545	36.1	SLC28A1	NM_004213.3	Intron	−362
rs7597224	0.189	0.019	0.85358	171	0.342	2	18829103	36.1	NT5C1B	NM_001002006.1	flanking_5UTR	−194784
rs12683783	−0.188	0.020	0.82119	171	0.126	9	86060628	36.1	SLC28A3	NM_022127.1	flanking_3UTR	−22284
rs10122651	−0.188	0.020	0.82119	171	0.468	9	86123704	36.1	SLC28A3	NM_022127.1	Intron	−5515
rs9294329	−0.187	0.021	0.82119	171	0.295	6	85958337	36.1	NT5E	NM_002526.1	flanking_5UTR	−258191
rs10780663	−0.186	0.022	0.82119	170	0.465	9	86120882	36.1	SLC28A3	NM_022127.1	Intron	−2693
rs9362176	−0.181	0.025	0.82119	171	0.152	6	86036174	36.1	NT5E	NM_002526.1	flanking_5UTR	−180354
rs4585823	−0.179	0.026	0.82119	171	0.132	9	86094005	36.1	SLC28A3	NM_022127.1	Intron	−884
rs12684950	−0.178	0.027	0.82119	171	0.155	9	86066751	36.1	SLC28A3	NM_022127.1	flanking_3UTR	−16161
rs556388	0.178	0.028	0.82119	171	0.447	8	1.03E+08	36.1	RRM2B	NM_015713.3	flanking_3UTR	−110177
rs10955282	0.179	0.028	0.82119	170	0.488	8	1.03E+08	36.1	RRM2B	NM_015713.3	flanking_3UTR	−39785
rs10780656	0.178	0.028	0.82998	171	0.196	9	86031222	36.1	SLC28A3	NM_022127.1	flanking_3UTR	−51690
rs1321742	−0.175	0.032	0.82119	170	0.444	6	85937908	36.1	NT5E	NM_002526.1	flanking_5UTR	−278620
rs501344	0.172	0.034	0.83550	171	0.327	8	1.03E+08	36.1	RRM2B	NM_015713.3	flanking_3UTR	−102158
rs3743162	0.171	0.035	0.82119	171	0.129	15	83231973	36.1	SLC28A1	NM_201651.1	Intron	−7
rs11885014	−0.173	0.035	0.82119	167	0.272	2	18810796	36.1	NT5C1B	NM_001002006.1	flanking_5UTR	−176477
rs9990999	0.170	0.037	0.83550	169	0.352	4	1.84E+08	36.1	DCTD	NM_001921.2	Intron	−8215
rs944664	−0.166	0.041	0.82119	171	0.444	6	85945694	36.1	NT5E	NM_002526.1	flanking_5UTR	−270834
rs4877824	0.165	0.042	0.82119	171	0.38	9	86045679	36.1	SLC28A3	NM_022127.1	flanking_3UTR	−37233
rs2251530	−0.163	0.044	0.82119	171	0.386	9	86002760	36.1	SLC28A3	NM_022127.1	flanking_3UTR	−80152
rs4507403	0.164	0.046	0.82119	166	0.41	4	1.84E+08	36.1	DCTD	NM_001012732.1	flanking_3UTR	−251819
rs6850978	0.159	0.050	0.82119	171	0.424	4	1.84E+08	36.1	DCTD	NM_001012732.1	flanking_3UTR	−238854

**Table 4:** Top 23 significant SNPs within or close to 9 pathway genes associated with AraC IC_50_ values (p<0.05).

SNP	r-value	p-value	q-value	N	MAF	Chr	Position	Genome Build	Gene Symbol	Gene	Location	Location Relative To Gene
rs9472236	0.240	0.003	0.99971	173	0.225	6	44314728	36.1	SLC29A1	NM_004955.1	flanking_3UTR	−4872
rs2037067	0.222	0.006	0.99971	172	0.471	4	183810578	36.1	DCTD	NM_001012732.1	flanking_3UTR	−237660
rs11999726	−0.219	0.006	0.99971	173	0.073	9	86104976	36.1	SLC28A3	NM_022127.1	intron	−516
rs7830150	−0.213	0.008	0.99971	173	0.187	8	103162269	36.1	RRM2B	NM_015713.3	flanking_3UTR	−123638
rs1614627	−0.196	0.015	0.99971	173	0.17	1	20819343	36.1	CDA	NM_001785.1	flanking_3UTR	−1360
rs2224211	0.192	0.017	0.99971	173	0.196	6	85619788	36.1	NT5E	NM_002526.1	flanking_5UTR	−596740
rs7874528	−0.183	0.023	0.99971	173	0.17	9	86020319	36.1	SLC28A3	NM_022127.1	flanking_3UTR	−62593
rs17414857	−0.182	0.023	0.99971	173	0.094	8	103139477	36.1	RRM2B	NM_015713.3	flanking_3UTR	−146430
rs4877839	−0.182	0.024	0.99971	173	0.12	9	86118829	36.1	SLC28A3	NM_022127.1	intron	−640
rs17343066	0.181	0.024	0.99971	173	0.36	9	86135893	36.1	SLC28A3	NM_022127.1	intron	−9416
rs12526113	−0.181	0.024	0.99971	173	0.33	6	85612351	36.1	NT5E	NM_002526.1	flanking_5UTR	−604177
rs2324753	−0.181	0.024	0.99971	173	0.33	6	85615181	36.1	NT5E	NM_002526.1	flanking_5UTR	−601347
rs12554292	−0.180	0.025	0.99971	173	0.135	9	86256385	36.1	SLC28A3	NM_022127.1	flanking_5UTR	−83152
rs1570311	−0.175	0.029	0.99971	173	0.161	6	86211870	36.1	NT5E	NM_002526.1	flanking_5UTR	−4658
rs4487548	−0.175	0.029	0.99971	173	0.161	6	86199130	36.1	NT5E	NM_002526.1	flanking_5UTR	−17398
rs9344525	−0.175	0.029	0.99971	173	0.161	6	86208157	36.1	NT5E	NM_002526.1	flanking_5UTR	−8371
rs6850978	0.171	0.034	0.99971	173	0.424	4	183809384	36.1	DCTD	NM_001012732.1	flanking_3UTR	−238854
rs9450270	−0.170	0.034	0.99971	173	0.187	6	86171485	36.1	NT5E	NM_002526.1	flanking_5UTR	−45043
rs3743162	0.170	0.034	0.99971	173	0.129	15	83231973	36.1	SLC28A1	NM_201651.1	intron	−7
rs1265138	0.165	0.042	0.99971	171	0.148	8	103269212	36.1	RRM2B	NM_015713.3	flanking_3UTR	−16695
rs1569040	0.163	0.043	0.99971	172	0.147	17	17246812	36.1	NT5M	NM_020201.3	flanking_3UTR	−55110
rs12500335	−0.162	0.044	0.99971	172	0.091	4	183944775	36.1	DCTD	NM_001012732.1	flanking_3UTR	−103463
rs11598702	−0.163	0.045	0.99971	171	0.246	10	104887975	36.1	NT5C2	NM_012229.2	intron	−1178

**Table 5:** 24 candidate SNPs with cis-regulation of pathway genes (p<0.0001).

SNP	Probeset	r-value (SNP vs Exp)	p-value (SNP vs Exp)	SNP	MAF	Chr	Position	Genome Build	Gene Symbol	Gene	Location	Location Relative to Gene
rs1319763	225044_at	0.6435	7.54E-33	rs1319763	0.22	17	37245316	36.1	NT5C3L	NM_052935.2	intron	−266
rs1046403	225044_at	0.6420	1.54E-32	rs1046403	0.219	17	37237346	36.1	NT5C3L	NM_052935.2	coding	[142/58]
rs4796712	225044_at	0.3728	2.69E-10	rs4796712	0.099	17	37240656	36.1	NT5C3L	NM_052935.2	coding	[77/12]
rs9344525	227486_at	−0.3034	3.91E-07	rs9344525	0.167	6	86208157	36.1	NT5E	NM_002526.1	flanking_5UTR	−8371
rs1570311	227486_at	−0.3034	3.91E-07	rs1570311	0.167	6	86211870	36.1	NT5E	NM_002526.1	flanking_5UTR	−4658
rs4487548	227486_at	−0.3034	3.91E-07	rs4487548	0.167	6	86199130	36.1	NT5E	NM_002526.1	flanking_5UTR	−17398
rs9344525	203939_at	−0.2821	2.59E-06	rs9344525	0.167	6	86208157	36.1	NT5E	NM_002526.1	flanking_5UTR	−8371
rs1570311	203939_at	−0.2821	2.59E-06	rs1570311	0.167	6	86211870	36.1	NT5E	NM_002526.1	flanking_5UTR	−4658
rs4487548	203939_at	−0.2821	2.59E-06	rs4487548	0.167	6	86199130	36.1	NT5E	NM_002526.1	flanking_5UTR	−17398
rs7277	201572_x_at	−0.2768	4.20E-06	rs7277	0.341	4	184048507	36.1	DCTD	NM_001012732.1	3UTR	[269/1038]
rs9344525	1553995_a_at	−0.2709	6.56E-06	rs9344525	0.167	6	86208157	36.1	NT5E	NM_002526.1	flanking_5UTR	−8371
rs1570311	1553995_a_at	−0.2709	6.56E-06	rs1570311	0.167	6	86211870	36.1	NT5E	NM_002526.1	flanking_5UTR	−4658
rs4487548	1553995_a_at	−0.2709	6.56E-06	rs4487548	0.167	6	86199130	36.1	NT5E	NM_002526.1	flanking_5UTR	−17398
rs4910907	201477_s_at	−0.2705	6.81E-06	rs4910907	0.167	11	4125427	36.1	RRM1	NM_001033.2	flanking_3UTR	−8745
rs9907244	225044_at	−0.2650	1.05E-05	rs9907244	0.455	17	37234426	36.1	NT5C3L	NM_052935.2	flanking_3UTR	−558
rs9344525	1553994_at	−0.2621	1.33E-05	rs9344525	0.167	6	86208157	36.1	NT5E	NM_002526.1	flanking_5UTR	−8371
rs1570311	1553994_at	−0.2621	1.33E-05	rs1570311	0.167	6	86211870	36.1	NT5E	NM_002526.1	flanking_5UTR	−4658
rs4487548	1553994_at	−0.2621	1.33E-05	rs4487548	0.167	6	86199130	36.1	NT5E	NM_002526.1	flanking_5UTR	−17398
rs2250159	203302_at	−0.2613	1.46E-05	rs2250159	0.369	9	85966866	36.1	SLC28A3	NM_022127.1	flanking_3UTR	−116046
rs1474500	201477_s_at	−0.2561	2.11E-05	rs1474500	0.166	11	4080688	36.1	RRM1	NM_001033.2	intron	−801
rs12990630	1553540_a_at	−0.2518	2.94E-05	rs12990630	0.082	2	18973412	36.1	NT5C1B	NM_001002006.1	flanking_5UTR	−339093
rs4910896	201477_s_at	−0.2492	3.58E-05	rs4910896	0.172	11	4118132	36.1	RRM1	NM_001033.2	flanking_3UTR	−1450
rs16845804	236703_at	0.2410	6.54E-05	rs16845804	0.221	4	72208285	36.1	DCK	NM_000788.1	flanking_3UTR	−92808
rs17271644	201572_x_at	−0.2376	8.30E-05	rs17271644	0.495	4	184046266	36.1	DCTD	NM_001012732.1	flanking_3UTR	−1972

### Functional Characterization of Candidate Genes

**Figure 4:**
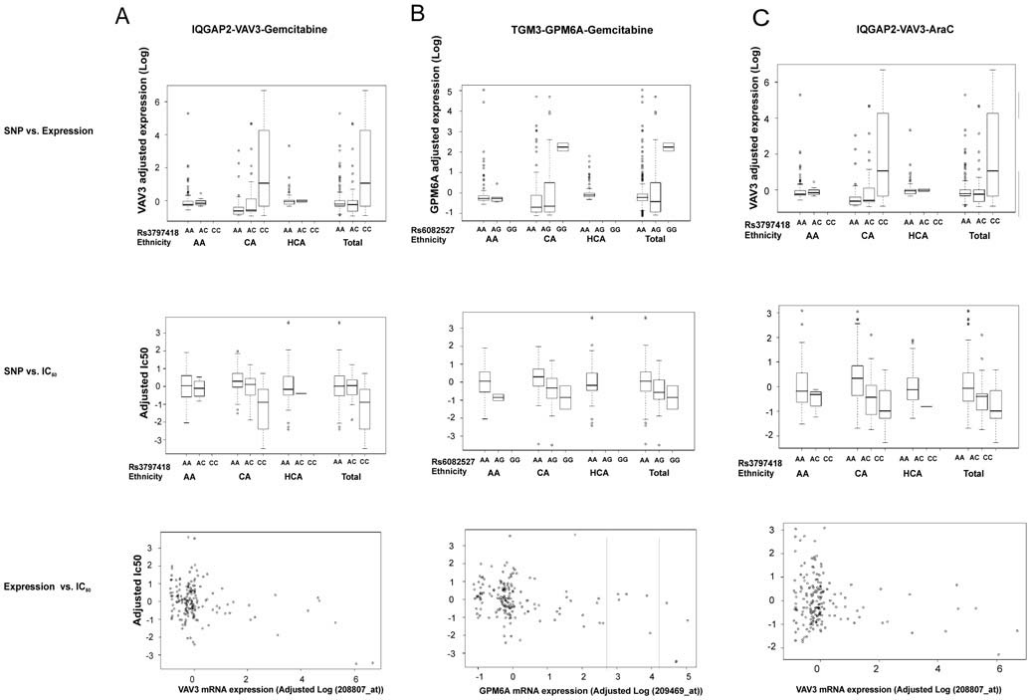
Association among SNPs in *IQGAP2* and *TGM3* with expression and gemcitabine or AraC cytotoxicity. (A) SNP rs3797418 (A/C) association with VAV3 expression, gemcitabine IC_50_ values and VAV3 expression association with gemcitabine IC_50_ in individual ethnic groups and all of the cell lines. (B) SNP rs6082527 (A/G) association with GPM6A gene expression, gemcitabine IC_50_ values and GPM6A gene expression association with gemcitabine IC_50_ values. (C). SNP rs3797418 (A/C) association with VAV3 expression, AraC IC_50_ values and VAV3 expression association with AraC IC_50_ in individual ethnic groups and all of the cell lines. Each dot represents one sample. Genotypes for each SNP are plotted against gene expression levels (upper panels) as well as gemcitabine or AraC IC_50_ values (middle panels). In the lower panel, correlations were determined between gene expression levels and gemcitabine or AraC IC_50_ values.

Our association studies were performed with human lymphoblastoid cell lines but the gene regulation is tissue specific [31]. Therefore, to investigate the impact of the SNPs that we had identified on gene expression and on gemcitabine and AraC cytotoxicity and to functionally validate our association results, we also studied tumor cell lines, in this case, two pancreatic cancer cell lines, to validate our association results. We performed siRNA knock down for three SNP-associated genes, including *VAV3*, *MGMT* that are associated with the SNP in *IQGAP2* and *GPM6A* which is associated with the SNP in *TGM3*, to determine whether genes that had expression associated with the two SNPs might influence drug IC_50_. We selected *VAV3* and *GPM6A* because each of these genes had two probesets associated with rs3797418 and rs6082527, respectively. VAV3 was also highly associated with gemcitabine and AraC IC_50_ while GPM6A gene expression was associated with gemcitabine IC_50_ (**Figure 4A and 4B**). As with *TGM3*, we also performed knock down of *GPM6A* for AraC. However, the association was most significant in the CA group, which could be due to differences in allele frequencies among the three ethnic groups studied. Expression of the third gene, *MGMT*, was also associated with both SNPs (rs3797418 with p = 1.22×10^−7^ and rs6082527 with p = 8.06×10^−8^) and with gemcitabine and AraC IC_50_ values (p = 5.49×10^−8^ and 1.85×10^−4^, respectively). We also included *IQGAP2* and *TGM3* in the siRNA knock down studies to determine whether the genes harboring these two SNPs might themselves have functional effects on gemcitabine or AraC cytotoxicity. Finally, we included a positive control siRNA, FKBP5, a gene that we had previously shown to be important for gemcitabine response [25].

**Figure 5:**
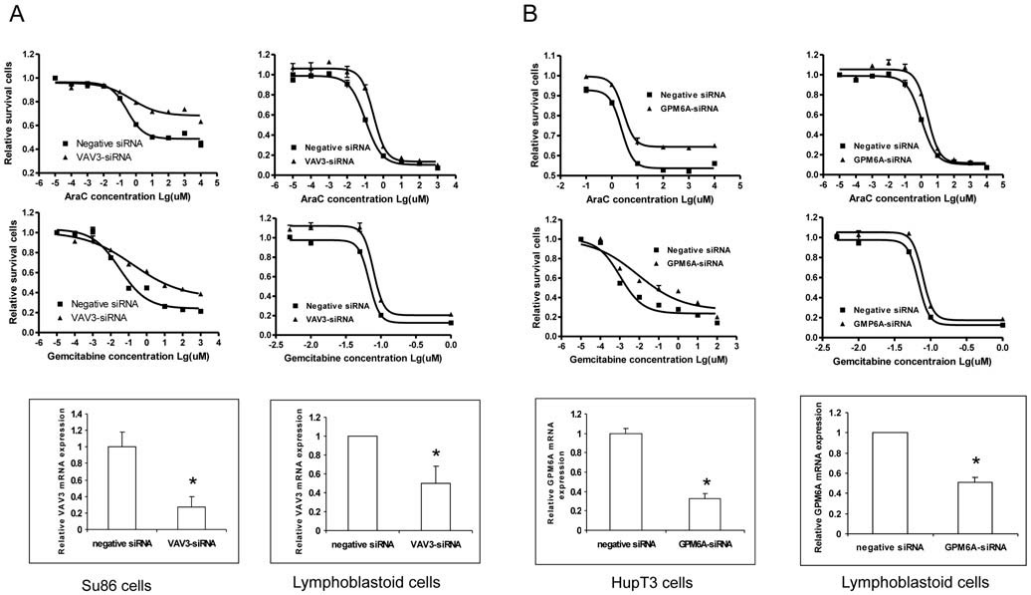
Functional characterization of candidate genes with siRNA knock down of VAV3 (A) and GPM6A (B). Knock down of VAV3 and GPM6A expression in human SU86 and Hup-T3 pancreatic cancer cell lines as well as lymphoblastoid cell lines showed increased resistance to gemcitabine and AraC after siRNA knock down as determined by MTS assays. Error bars represent SEM values for 3 independent experiments. Quantitative RT-PCR was performed to assess VAV3 and GPM6A gene expression levels after knock down with specific siRNAs. Results are expressed as % of control. Error bars represent SEM values for 3 independent experiments. *  = p<0.05.

Specific and negative control siRNAs were transiently transfected into human SU86 or Hup-T3 pancreatic cancer cell lines, cell lines selected on the basis of endogenous expression level of genes of interest, followed by gemcitabine or AraC cytotoxicity assays. We selected pancreatic cancer cell lines because gemcitabine is the standard of care for the therapy of pancreatic cancer. Down regulation of VAV3 in SU86 cells significantly desensitized the cells to gemcitabine and AraC (**Figure 5A**), consistent with the result of the association study (gemcitabine p = 9.14E-10 and AraC p = 1.24E-11). Down regulation of GPM6A in HupT3 cell line also resulted in desensitization to gemcitabine (p = 1.35E-07), an observation consistent with our association study results for GPM6A gene expression and gemcitabine IC_50_ (**Figure 5B**). Interestingly, although GPM6A expression was not significantly associated with AraC response, the knock down experiments showed a similar effect for AraC (p = 3.66E-12) (**Figure 5B**). We also performed similar experiments of knock down of these two genes using selected lymphoblastoid cell lines and it showed similar results as in the tumor cell lines (**Figure 5A** right panel, AraC p = 0.0074, gemcitabine p = 0.0085; **Figure 5B** right panel, AraC p = 0.001, gemcitabine p = 0.002). However, down-regulation of MGMT did not alter either gemcitabine or AraC cytotoxicity. Furthermore, knock down of IQGAP2 and TGM3 themselves, did not have significant effect on gemcitabine or AraC cytotoxicity, an observation indicating that the genes harboring these two SNPs might not have a direct effect on cytotoxicity for the two drugs, and that rs3797418 and rs6082527 might just be markers for gemcitabine and/or AraC cytotoxicity.

### Characterization of SNPs in *IQGAP2* and *TGM3*

**Figure 6:**
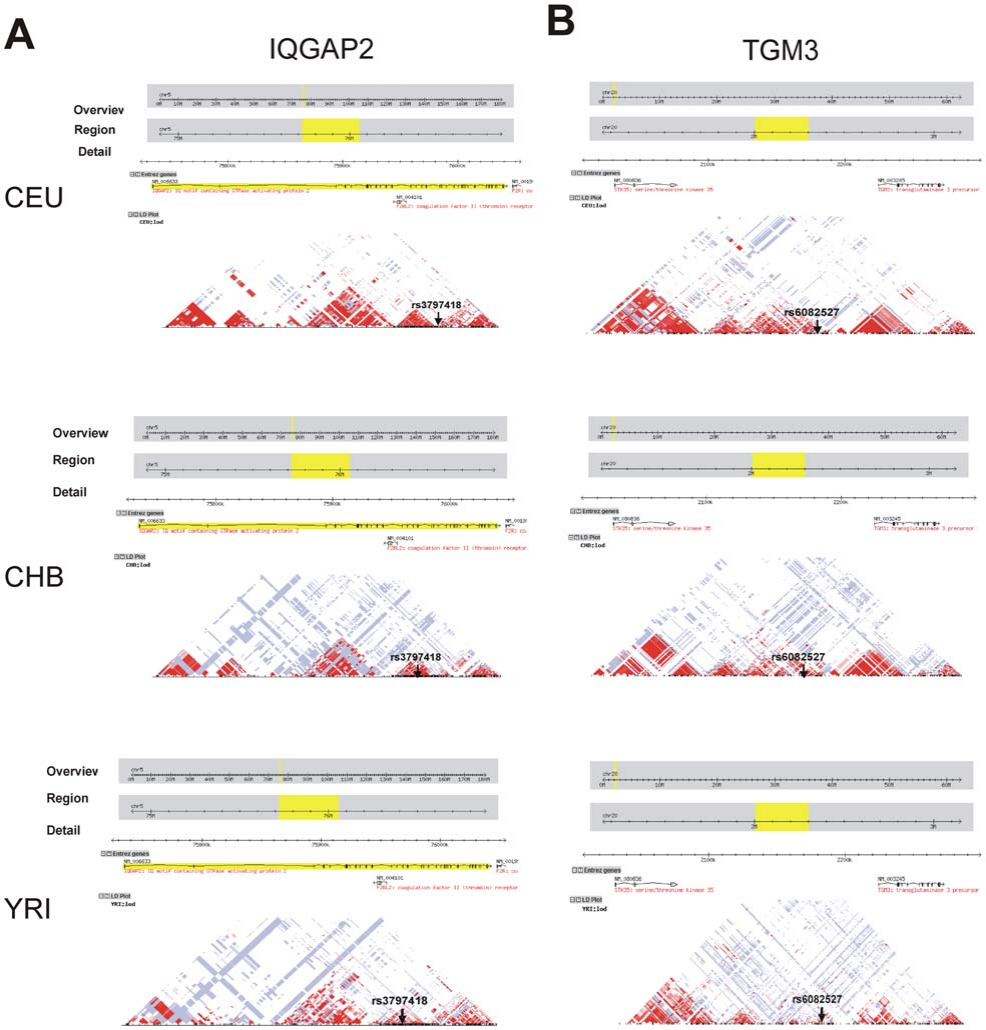
Linkage disequilibrium within ∼300 Kb surrounding the rs3797418 and rs6082527 SNPs. Red indicates combinations where D′ = 1 and linkage of disequilibrium (LOD) ≥2; light red, combinations where D′<1 and LOD ≥2; red squares, combinations where D′ = 1 and LOD <2; white squares, combinations with D′<1 and LOD <2. SNPs are arranged in order from 5′ to 3′ in each gene, as shown in the gene structure above each plot.

In order to further characterize the two SNPs (rs3797418 and rs6082527) in *IQGAP2* and *TGM3*, we also performed genotype-phenotype correlation studies with the expression of these two genes, i.e., we determined the possible cis-association of the SNPs with IQGAP2 and TGM3 expression. However, neither SNP was associated with the expression of either IQGAP2 or TGM3, indirectly supporting the results of the *IQGAP2* and *TGM3* knock down experiments. We then determined LD patterns for 500 kb regions around each of the two SNPs using the HapMap data for each ethnic group. As shown in **Figure 6**. LD patterns surrounding the two SNPs differed among the three ethnic groups. No SNPs within the 500 kb region around rs3797418 were in high LD with rs3797418. One SNP, rs6047696 in an intergenic region, was in moderate LD with rs6082527 in the CEPH population (r^2^ = 0.682, D′ = 0.826). In addition, rs12479538, located in an intron of *STK35*, a serine/threonine kinase 35, was in complete linkage disequilibrium with rs6082527 in the Han Chinese population (r^2^ = 1, D′ = 1). The p-value for the association between the rs6082527 SNP and gemcitabine IC_50_ was 0.006, which was not significant after correction for multiple comparisons. Unfortunately, the rs12479538 SNP was not on the 550K SNP array. Obviously, the association of the two SNPs in *IQGAP2* and *TGM3* with gemcitabine or AraC response and with the expression of other genes might result from the effects of either known or unknown polymorphisms in tight linkage disequilibrium with these two SNPs.

Since these two SNPs, rs3797418 (A/C) and rs6082527 (A/G), were located in the intron of *IQGAP2* and the 5′-FR of *TGM3*, respectively, we determined the relationship among genes associated with these two SNPs. Ingenuity Pathway analysis was performed by mapping the 10 and 8 unique genes that were significantly associated with rs3797418 in *IQGAP2* for gemcitabine and AraC, respectively. Results of the Ingenuity Pathway Analysis showed a network that connected all of the 10 genes centered on ERK and TNF for gemcitabine and all of the 8 genes centered on TNF, ERK, TP53 for AraC (**Figure S1**). However, since only 5 genes were identified to be associated with rs6082527 through our analysis, no network was identified when we analyzed genes that were associated with this SNP.

## Discussion

Variation in response to chemotherapeutic agents is a common phenomenon in the clinic. Many factors can contribute to this variation. However, genetic variation is one of the major factors that play an important role in determining response to drugs. Therefore, it is important to identify SNPs that might be used as predictive markers for drug response. In a previous study, we identified candidate genes for which variation in expression levels were associated with variation in response to gemcitabine and AraC using a lymphoblastoid cell line model system [25]. In the current set of experiments, we expanded our study to include genome-wide SNPs to test the hypothesis that SNPs across the genome might contribute significantly to variation in response to two cytidine analogues, gemcitabine and AraC. Lymphoblastoid cell lines with genome-wide SNP and expression data have been used successfully to identify pharmacogenomic candidates associated with response to other chemotherapeutic agents [26], [32]. The advantage of using this cell-based model system is that we have obtained different types of high throughput genomic and transcriptomic data, which makes it possible to test the hypothesis that genetic variation might contribute to variation in drug response, in this case, two cytidine analogues [18], [19], [33], [34]. Many previous pharmacogenetic studies for these two drugs were focused on the gemcitabine bioactivation and metabolism pathway [35], [36]. For example, SNPs identified in genes encoding ribonucleotide reductase (RRM1) and cytidine deaminase (CDA) were found to be associated with gemcitabine chemosensitivity in the NCI60 cell lines or with active gemcitabine plasma metabolites levels [37]–[39]. Those findings provided the initial evidence that genetic variation might contribute to the variation in cytidine analogue response.

In the present study, we performed a genome-wide association study with 171 lymphoblastoid cell lines for which we obtained genome-wide SNPs by use of Illumina 550K SNP chips, as well as, expression array data obtained with Affymetrix U133 Plus 2.0 GeneChips. Those data made it possible for us to take a comprehensive approach in which we combined genotype, expression and cytotoxicity data for the two cytidine analogues (**Figure 3**) to identify SNPs that might contribute to drug sensitivity through their impact on the regulation of gene expression (**Tables 1** and **2**). However, there were only 49 SNPs among the top significant SNPs for gemcitabine and AraC that were common for both drugs, indicating even though the two drugs share common mechanisms and the same metabolic pathway, there are differences between these two drugs. This stepwise analysis helped us to narrow our focus to two SNPs associated with both expression and drug cytotoxicity. Those two SNPs, rs3797418 and rs6082527, were located, respectively, in an intron of *IQGAP2* and the 5′-FR of *TGM3*. To further understand the biology responsible for the association of rs3797418 and rs6082527 with gene expression and drug cytotoxicity, we performed knock down of selected genes with expression that was associated with these two SNPs. Knock down of VAV3 and GPM6A in pancreatic cancer cell lines desensitized the cells to gemcitabine and AraC (**Figure 5A and 5B**). VAV3 is a member of the VAV family that has guanine nucleotide exchange activity toward small GTP-binding proteins. VAV3 is a known proto-oncogene that can be involved in tumorgenesis [40]–[42]. As shown in our network analysis (**Figure S1**), VAV3 can be involved in multiple signaling pathways. Although the mechanism responsible for VAV3 involvement in gemcitabine and AraC response is unclear, given the multiple roles of VAV3 in cancer development, it was not surprising that this gene might contribute to variation in response to chemotherapeutic agents. GPM6A, also known as M6a, is a transmembrane glycoprotein that belongs to the myelin protolipid protein (PLP) family. This gene is expressed mainly in nervous system, and recent studies have suggested the importance of GPM6A in processes involved in neural development such as neurite extension, survival and differentiation [43]–[45]. GPM6A also interacts with a number of G protein coupled receptors and downstream signaling pathways [46]. One recent report suggested that GPM6A might function as a chaperone in cancer cells as part of global profiling of the cell surface proteome for cancer cells [47]. Although there is limited information with regard to the involvement of this gene in tumorgenesis and drug resistance, our results suggest a possible role for *GPM6A* in response to cytidine analogue antineoplastic agents.

Although the two SNPs in *IQGAP2* and *TGM3* that we studied were associated with multiple downstream genes with expression that was also associated with gemcitabine or AraC cytotoxicity, we did not identify an association between these two SNPs and either IQGAP2 and TGM3 gene expression, nor did we observe an influence of IQGAP2 and TGM3 on gemcitabine or AraC cytotoxicity after knock down of these two genes.

*IQGAP2* is approximately 300 kb and *TGM3* is about 45 kb in length. **Figure 6** shows the haplotype structure within ∼300 kb surrounding the rs3797418 and rs6082527 SNPs. Linkage analysis was also performed with HapMap data sets in different ethnic groups. However, one of the SNPs linked to rs6082527 was not associated with gemcitabine IC_50_, and the other was not present on the 550K SNP array. Therefore, the effect of the two SNPs in *IQGAP2* and *TGM3* on drug response and other gene expression might result from the effects of either known or unknown polymorphisms linked to those two SNPs.

In summary, we have used a data-rich cell-based model system to perform association studies of gemcitabine and AraC cytotoxicity with genome-wide SNP and expression array data. Use of this approach enabled us to identify SNPs that might be markers for cytidine analogue sensitivity and genes that might play an important role in variation in response to these drugs. There are also limitations associated with the use of these cell lines, as described by Choy et. al [48]. The potential confounding factors besides genetic effect could include cell growth rate, EBV transformation and ATP condition. However, that same paper stated that “RNA levels are predominantly correlated to inter-individual differences in EC50. Much less of the correlation between RNA and EC50s reflects intra-individual variation”. That conclusion supports the hypothesis that inter-individual variation in RNA levels due to genetic variation may contribute to variation in drug response phenotype. One limitation of the use of these cell lines is the possibility that EBV transformation might influence drug sensitivity and/or expression profiles, so we might miss some genes of importance for the drug response phenotype, either because they are not expressed in these cell lines or, after transformation, are down-regulated. There is also evidence that gemcitabine and doxorubicin can induce the lytic form of EBV transformed cells [49], [50]. However, we and other groups have demonstrated success with the use of this type of cell line in previous pharmacogenomic studies [25], [26], [51], and functional characterization of the genes identified during the present study supports the feasibility of this approach. The results of the present study enhance our understanding of the role of genetic variation in variation in gemcitabine and AraC sensitivity and resistance and may help to identify mechanisms involved in the actions of these drugs. In addition, these SNPs and genes can now potentially be tested in the clinical setting and, if confirmed, they could significantly enhance our ability to individualize treatment with these two drugs.

## Materials and Methods

### Cell Line

174 lymphoblastoid cell lines from 60 Caucasian-American (CA), 54 African-American (AA) and 60 Han Chinese-American (HCA) (sample sets HD100CAU, HD100AA, and HD100CHI) subjects were purchased from the Coriell Cell Repository (Camden, NJ). All of these cell lines had been obtained and anonymized by the National Institute of General Medical Sciences prior to deposit, and all subjects had provided written consent for the use of their DNA and cells for experimental purposes. Human SU86 and Hup-T3 pancreatic cancer cell lines were gifts from Dr. Daniel D. Billadeau, Mayo Clinic.

### siRNA

The siRNA duplexes used in the knock down studies were purchased from the QIAGEN Inc. (Valencia, CA).

### Drugs and Cell Proliferation Assays

Gemcitabine was provided by Eli Lilly (Indianapolis, IN). AraC was purchased from Sigma-Aldrich (St. Louis, MO). Cytotoxicity assays with the lymphoblastoid cell and tumor cell lines were performed with the CellTiter 96® AQ_ueous_ Non-Radioactive Cell Proliferation Assay (Promega Corporation, Madison, WI) as previously described [25]. Estimation of the IC_50_ phenotype (effective dose that kills 50% of the cells) was calculated using a four parameter logistic model. Of the 174 cell lines, cytotoxicity curves for two cell lines were removed from further analysis due to possible experimental error.

### Gene Expression

Expression array data were obtained for all 174 lymphoblastoid cell lines as previously described [25]. Specifically, biotin-labeled cRNA, produced by *in vitro* transcription, was hybridized to Affymetrix Human Genome U133 Plus 2.0 GeneChips. Each of these GeneChip contains 54,613 probesets. Normalization of the expression array data was performed using GCRMA [52], [53].

### Genome-Wide SNP Analysis

Illumina HumanHap 550K BeadChips were used to obtain genome-wide SNP data for all 174 cell lines. Specifically, each DNA sample was genotyped using the Illumina version 3 BeadChip, which assays 561,278 single nucleotide polymorphisms (SNPs). All genotyping was conducted at the Genotype Shared Resource (GSR) at the Mayo Clinic, Rochester, MN. SNPs with call rates <0.95 were excluded, as were DNA samples with call rates <0.95. The call rate for rs3797418 and rs6082527 SNPs were 100% for all cell lines. One African American sample was removed due to a call rate <0.95, resulting in 173 cell lines with genotypic data and 171 cell lines with both genotypic data and phenotypic data (gemcitabine IC_50_ values)

### Transient Transfection and RNA Interference

Human SU86 and HupT3 pancreatic cancer cell lines were used to perform the siRNA studies. The Hiperfect transfection reagent (QIAGEN) was used for siRNA reverse transfection. Specifically, cells were seeded into 96-well plates and were mixed with siRNA-complex, consisting of 5 nM of specific or negative control siRNA (QIAGEN), and the Hiperfect transfection reagent. The lymphoblastoid cell lines were transfected with control siRNA and specific siRNA for GPM6A and VAV3 using the electroporation (Nucleofector® Lonza Walkersville Inc., Walkersville, MD). Specifically, 1.25×10^6^ cells suspended in Cell Line Nucleofector Solution V (Lonza Walkersville Inc.) were mixed with 2 µM control or specific siRNA before the electroporation.

### Quantitative Real-Time Reverse Transcription-PCR

Total RNA was isolated from cultured cells with the Qiagen RNeasy kit (QIAGEN Inc. Valencia, CA), followed by QRT-PCR performed with the 1-step, Brilliant SYBR Green QRT-PCR master mix kit (Stratagene, La Jolla, CA). Specifically, primers purchased from Qiagen were used to perform QRT-PCR using the Stratagene Mx3005P™ Real-Time PCR detection system (Stratagene). All experiments were performed in triplicate with β-actin as an internal control. Reverse transcribed Universal Human reference RNA (Stratagene) was used to generate a standard curve. Control reactions lacked RNA template.

### Statistical Methods

Quality control for the Illumina genotypes was performed before performing the association study. SNPs that deviated from HWE (minimum exact test for HWE [54], [55] and stratified test for HWE [56] p-values <0.001), with call rates <95% or SNPs with MAFs <5% were removed from the analysis. Subjects with call rates <95% were also removed from all SNP analyses. No effects for SNPs were seen with the Illumina platforms. The plot for the most significant SNPs are shown in **Figure S2**. Next, population stratification was assessed. Because we studied cell lines chosen to represent common human genetic diversity, multiple races/ethnic groups were represented in the lymphoblastoid cell lines. Therefore, we used the method developed by Price et al. [57] to adjust for population stratification. This method uses an eigen analysis to detect and adjust for population stratification. Within each race, eigen analysis was completed, with the top five eigenvectors saved. Using the five eigenvectors within each race, individual genotypes were adjusted using the model 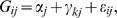 with 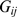 representing the genotype for the i^th^ cell line in racial group *j* (*j* = 1, 2, 3), 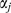 the race effect for race *j*, and 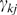 the *k* ^th^ eigenvector, *k* = 1, 5, effect for race *j*. Similarly, the log transformed IC_50_ values, were also adjusted for race using the five eigenvectors, in addition to gender. Pearson correlation coefficients were then computed using the adjusted genotypes and IC_50_ variables. False Discovery q-values [58], [59] were also computed for each test. Similar sets of analyses were also performed for the association of IC_50_ with mRNA expression and SNPs with mRNA expression. Pairwise LD was estimated using r-squared statistics and were displayed graphically using the Haploview software [60].

### Integrative Genomic Analysis

To integrate genotype, expression and drug cytotoxicity data, we used an approach similar to “genomic convergence” [61]–[63], as depicted graphically in **Figure 2**. We used a less stringent cut off for p-values used to identify the initial set of SNPs that were associated with IC_50_ to avoid missing possible candidates with biological significance. First, we identify SNPs associated with IC_50_ (p-values <0.001). One of the functional mechanisms by which SNPs might influence cytotoxicity is by regulating transcription in either a cis (SNP within or near the gene) or trans- (SNP not within or near the gene) manner. Therefore, we determined the expression probesets that were associated with these identified SNPs (p-values <10^−6^). Next, to determine whether the expression probesets associated with these SNPs were also associated with IC_50_ values for gemcitabine and AraC, we correlated these expression probesets with IC_50_ for both drugs with a p<0.001. A set of genes was then selected to test functionally for their possible biological relevance with regard to drug response.

### Bioinformatics Pathway Analysis

To further understand biological interactions between the downstream genes, pathway analysis was performed on genes that had significant associations with the two SNPs and with phenotype. In the case of gemcitabine, SNP rs3797418 present in the intronic region of *IQGAP2* showed significant associations with 14 probesets during the SNP-expression analysis; and these same 14 probesets also had significant correlations with IC_50_ phenotype. Therefore, these14 probesets that mapped to 10 unique genes were used to perform pathway analysis. A similar analysis was performed with AraC using the 8 genes that were significantly associated with the SNP rs3797418 to perform the pathway analysis. SNP rs6082527 close to *TGM3* gene was associated with 6 probesets that showed significant correlations with IC_50_ phenotype. Therefore, pathway analysis was also performed for the 6 probesets that mapped to 5 genes to identify major pathways that might be involved with the downstream effects of this SNP. Ingenuity software was used to perform the pathway analysis. This software consists of a curated database and several analysis tools to obtain pathways associated with a set of genes. Ingenuity calculates p-value for the probability of finding a set of genes within a given pathway. Fisher's exact test is used to calculate the p-values.

## Supporting Information

Table S1 Top polymorphism associated with gemcitabine cytotoxicity (IC50 values). 492 significant SNPs were associated with gemcitabine IC50 values with p-values <0.001.
(0.09 MB XLS)

Table S2 Top candidates for association between 550K genome-wide SNPs and AraC cytotoxicity. 553 significant SNPs were associated with AraC IC50 values with p-values <0.001.
(0.10 MB XLS)

Table S3 Top SNPs that were significantly associated with both gemcitabine and AraC (p<0.001).
(0.05 MB XLS)

Table S4 Top candidates for association between 492 SNPs and whole gene expression array data (54,000 probe sets). 100 SNPs among 492 located within or close to 78 genes were associated with 296 expression probe sets (220 unique genes) with p-values <10−6.
(0.19 MB XLS)

Table S5 Top candidates for association between 553 SNPs and whole gene expression data (54,000 probesets). 140 SNPs among 553 SNPs located within or close to 843 probe sets (563 unique genes) with p-values <10−6.
(0.33 MB XLS)

Table S6 Genes within the gemcitabine/AraC transport, metabolism and target pathway.
(0.02 MB PDF)

Figure S1 Network analysis. Genes that were associated with rs3797418, an intron SNP in IQGAP2, were used to perform network analysis using Ingenuity Pathway Analysis. Dotted line indicates an indirect connection and solid lines indicate a direction interaction between genes.
(0.32 MB PDF)

Figure S2 Illumina intensity plot for top candidate SNPs.
(0.13 MB PDF)
