# Supplementary material for: Gemcitabine and Arabinosylcytosin Pharmacogenomics: Genome-Wide Association and Drug Response Biomarkers
Source: PLoS One. 2009 Nov 9;4(11):e7765. doi: 10.1371/journal.pone.0007765 (PMC2770319; doi:10.1371/journal.pone.0007765)
Supplement: Table S6 — Genes within the gemcitabine/AraC transport, metabolism and target pathway. (0.02 MB PDF) [file pone.0007765.s006.pdf]

| HUGO Name | Gene Name | Chromosomal Location |
|---|---|---|
| *DCK* | Deoxycytidine kinase | 4q13.3-q21.1 |
| *CMPK* | Cytidylate kinase | 1p32 |
| *CDA* | Cytidine deaminase | 1p36.2-p35 |
| *DCTD* | dCMP deaminase | 4q35.1 |
| *RRM1* | Ribonucleotide reductase M1 | 11p15.5 |
| *RRM2* | Ribonucleotide reductase M2 | 2p25-p24 |
| *RRM2B* | Ribonucleotide reductase M2B (TP53 inducible) | 8q23.1 |
| *SLC28A1* | *Solute carrier family 28 member 1* | *15q25-26* |
| *SLC28A2* | *Solute carrier family 28 member 2* | *15q15* |
| *SLC28A3* | *Solute carrier family 28 member 3* | *9q22.2* |
| *SLC29A1* | *Solute carrier family 29 member 1* | *6q21.1-p21.2* |
| *SLC29A2* | *Solute carrier family 29 member 2* | *11q13* |
| *NT5C1A* | *5′-nucleotidase, Cytosolic IA* | *1p34.3-p33* |
| *NT5C1B* | *5′-nucleotidase, Cytosolic IB* | *2p24.2* |
| *NT5C2* | 5′-nucleotidase, Cytosolic II | 10q24.32-q24.33 |
| *NT5C3* | 5′-nucleotidase, Cytosolic III | 7p14.3 |
| *NT5E* | 5'-nucleotidase, Ecto (CD73) | 6q14-q21 |
| *NT5C3L* | 5'-nucleotidase, Cytosolic III-like | 17q21.2 |
| *NT5C* | 5′-nucleotidase, cytosolic | 17q25.1 |
