# Supplementary figures and images for: Gemcitabine and Arabinosylcytosin Pharmacogenomics: Genome-Wide Association and Drug Response Biomarkers
Source: PLoS One. 2009 Nov 9;4(11):e7765. doi: 10.1371/journal.pone.0007765 (PMC2770319; doi:10.1371/journal.pone.0007765)

## References

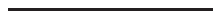

Figure S1B

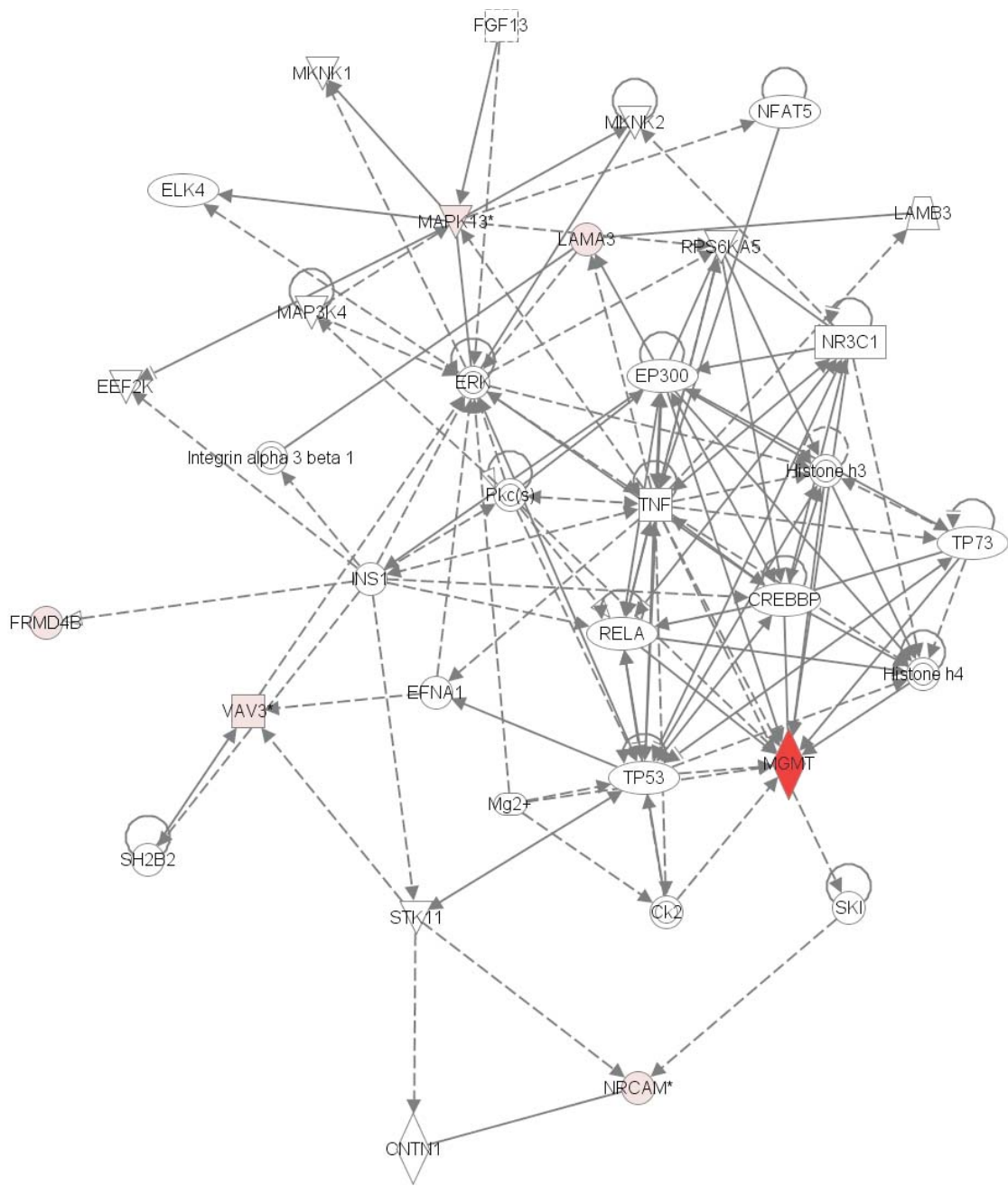

Supplement: Figure S1 — Network analysis. Genes that were associated with rs3797418, an intron SNP in IQGAP2, were used to perform network analysis using Ingenuity Pathway Analysis. Dotted line indicates an indirect connection and solid lines indicate a direction interaction between genes. (0.32 MB PDF) [file pone.0007765.s007.pdf]
