# Supplementary material for: Gemcitabine and Arabinosylcytosin Pharmacogenomics: Genome-Wide Association and Drug Response Biomarkers
Source: PLoS One. 2009 Nov 9;4(11):e7765. doi: 10.1371/journal.pone.0007765 (PMC2770319; doi:10.1371/journal.pone.0007765)
Supplement: Figure S2 — Illumina intensity plot for top candidate SNPs. (0.13 MB PDF) [file pone.0007765.s008.pdf]

Figure S2

<u>Intensity plots of rs3797418 nd rs6082527.</u> The plots are generated using Illumina Bead studio software. The plots show the data from Illumina 550K chip SNP assay.

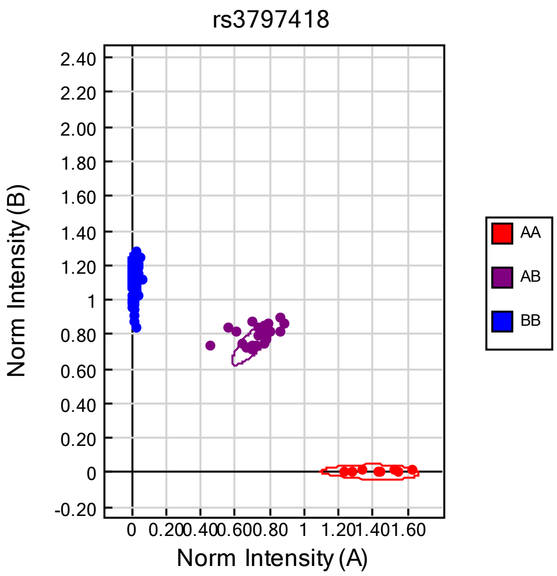

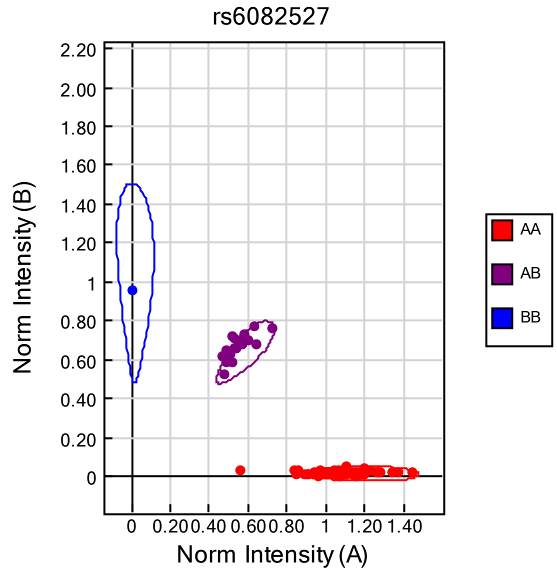

Intensity plots of top 5 SNPs from genome-wide SNP association study with gemcitabine IC_{50} value.

The plots are generated using Illumina Bead studio software. The plots show the data from Illumina 550K chip SNP arrays.

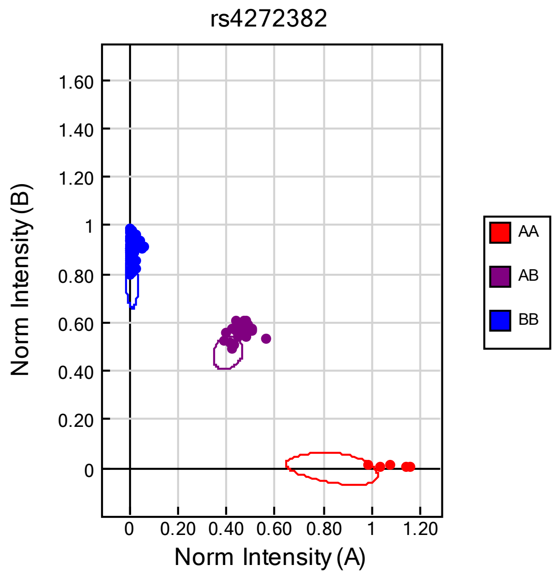

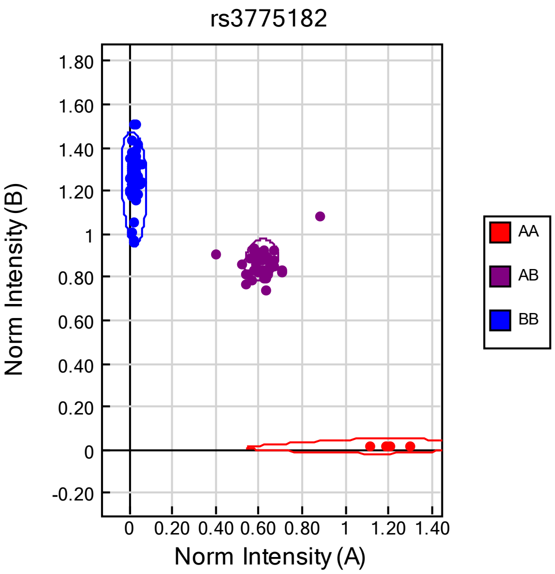

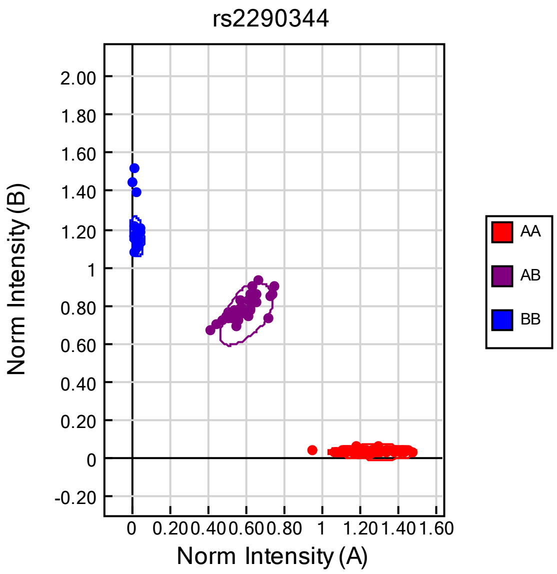
rs2290344
Norm Intensity (B)
Norm Intensity (A)
2.00
1.80
1.60
1.40
1.20
1
0.80
0.60
0.40
0.20
0
-0.20
0
0.20
0.40
0.60
0.80
1
1.20
1.40
1.60
AA
AB
BB

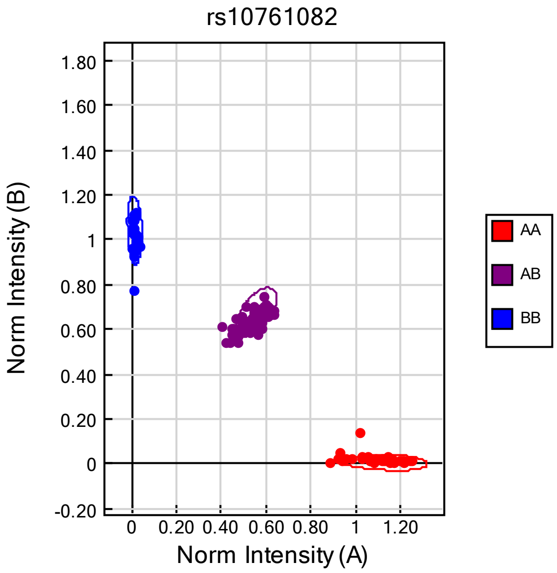
rs10761082
Norm Intensity (B)
Norm Intensity (A)
1.80
1.60
1.40
1.20
1
0.80
0.60
0.40
0.20
0
-0.20
0
0.20
0.40
0.60
0.80
1
1.20
AA
AB
BB

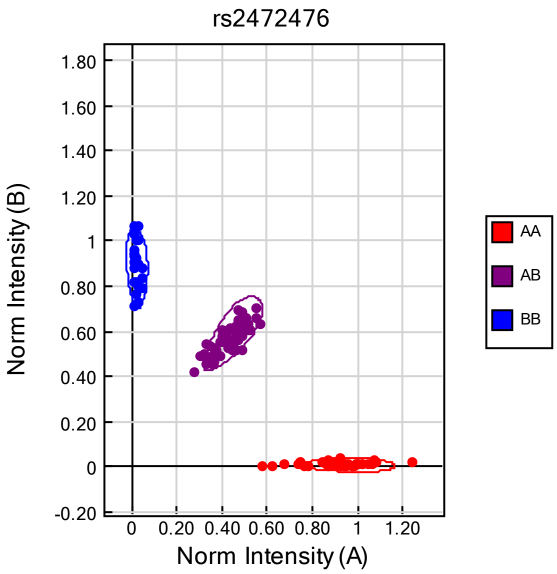

Intensity plots of top 5 SNPs from genome-wide SNP association study with AraC IC_{50} value. The plots are generated using Illumina Bead studio software. The plots show the data from Illumina 550K chip SNP arrays.

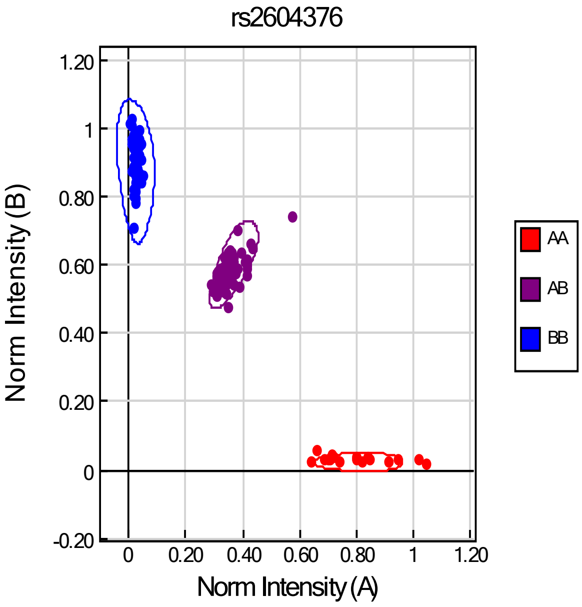

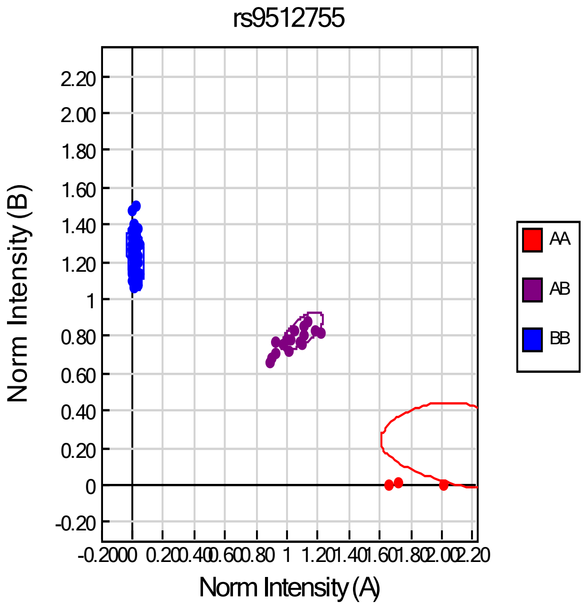
rs9512755
Norm Intensity (B)
Norm Intensity (A)
AA
AB
BB

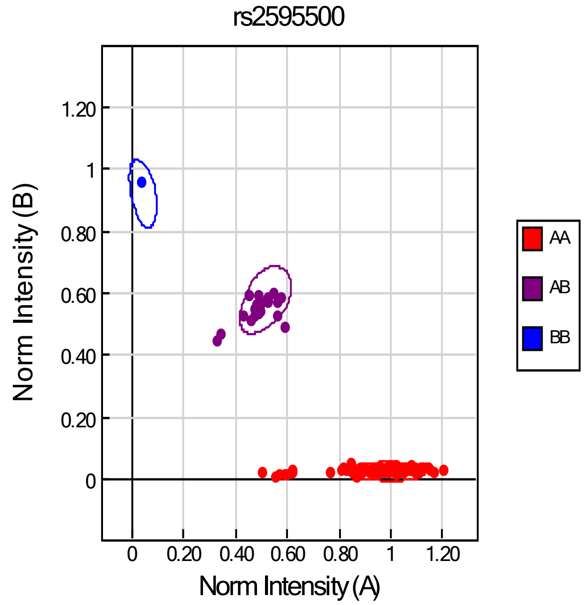
rs2595500
Norm Intensity (B)
Norm Intensity (A)
AA
AB
BB

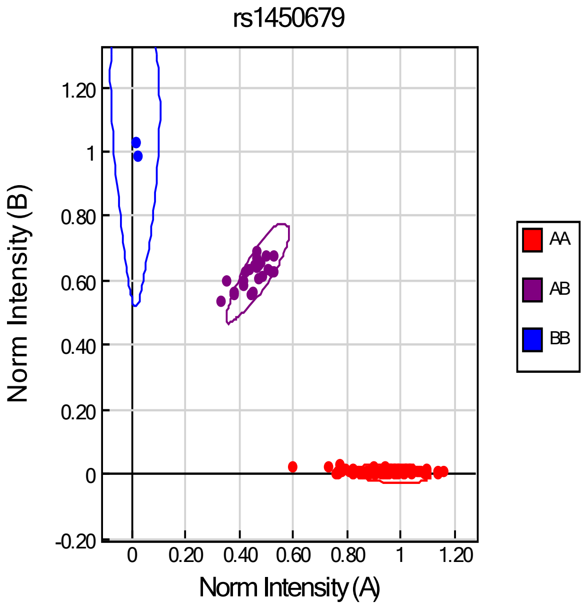
rs1450679
Norm Intensity (B)
1.20
1
0.80
0.60
0.40
0.20
0
-0.20
0
0.20
0.40
0.60
0.80
1
1.20
Norm Intensity (A)
AA
AB
BB

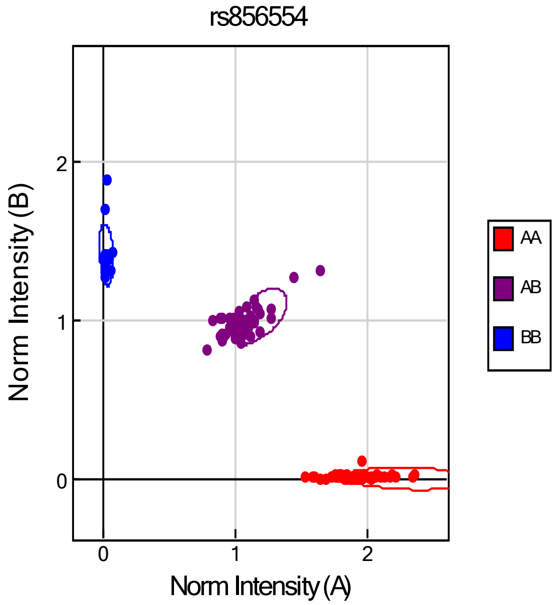
rs856554
Norm Intensity (B)
2
1
0
0
1
2
Norm Intensity (A)
AA
AB
BB
